# Cerebrospinal fluid lipoproteins inhibit α-synuclein aggregation by interacting with oligomeric species in seed amplification assays

**DOI:** 10.1186/s13024-023-00613-8

**Published:** 2023-04-01

**Authors:** Giovanni Bellomo, Silvia Paciotti, Luis Concha-Marambio, Domenico Rizzo, Anna Lidia Wojdaƚa, Davide Chiasserini, Leonardo Gatticchi, Linda Cerofolini, Stefano Giuntini, Chiara Maria Giulia De Luca, Yihua Ma, Carly M. Farris, Giuseppe Pieraccini, Sara Bologna, Marta Filidei, Enrico Ravera, Moreno Lelli, Fabio Moda, Marco Fragai, Lucilla Parnetti, Claudio Luchinat

**Affiliations:** 1 Laboratory of Clinical Neurochemistry, Section of Neurology, Department of Medicine and Surgery, University of Perugia, Piazzale Lucio Severi 1/8, 06132 Perugia, Italy; 2 R&D Unit, Amprion Inc, 11095 Flintkote Av., San Diego, San Diego, CA 92121 USA; 3 Magnetic Resonance Center (CERM), University of Florence, Via Luigi Sacconi 6, 50019 Sesto Fiorentino, Italy; 4 Department of Chemistry “Ugo Schiff”, University of Florence, Via Della Lastruccia 3, 50019 Sesto Fiorentino, Italy; 5 Section of Physiology and Biochemistry, Department of Medicine and Surgery, University of Perugia, Piazzale Lucio Severi 1/8, 06132 PerugiaPerugia, Italy; 6 Consorzio Interuniversitario Risonanze Magnetiche Metallo Proteine (CIRMMP), Via Luigi Sacconi 6, 50019 Sesto Fiorentino, Italy; 7 Division of Neurology 5 and Neuropathology, Fondazione IRCCS Istituto Neurologico Carlo Besta, Via Celoria 11, 20133 Milan, Italy; 8 Department of Health Sciences, CISM Mass Spectrometry Centre, University of Florence, Viale Gaetano Pieraccini 6, 50139 Florence, Italy

**Keywords:** α-synuclein, Lipoproteins, Cerebrospinal fluid, Seed amplification assays, RT-QuIC

## Abstract

**Background:**

Aggregation of α-synuclein (α-syn) is a prominent feature of Parkinson’s disease (PD) and other synucleinopathies. Currently, α-syn seed amplification assays (SAAs) using cerebrospinal fluid (CSF) represent the most promising diagnostic tools for synucleinopathies. However, CSF itself contains several compounds that can modulate the aggregation of α-syn in a patient-dependent manner, potentially undermining unoptimized α-syn SAAs and preventing seed quantification.

**Methods:**

In this study, we characterized the inhibitory effect of CSF milieu on detection of α-syn aggregates by means of CSF fractionation, mass spectrometry, immunoassays, transmission electron microscopy, solution nuclear magnetic resonance spectroscopy, a highly accurate and standardized diagnostic SAA, and different in vitro aggregation conditions to evaluate spontaneous aggregation of α-syn.

**Results:**

We found the high-molecular weight fraction of CSF (> 100,000 Da) to be highly inhibitory on α-syn aggregation and identified lipoproteins to be the main drivers of this effect. Direct interaction between lipoproteins and monomeric α-syn was not detected by solution nuclear magnetic resonance spectroscopy, on the other hand we observed lipoprotein-α-syn complexes by transmission electron microscopy. These observations are compatible with hypothesizing an interaction between lipoproteins and oligomeric/proto-fibrillary α-syn intermediates. We observed significantly slower amplification of α-syn seeds in PD CSF when lipoproteins were added to the reaction mix of diagnostic SAA. Additionally, we observed a decreased inhibition capacity of CSF on α-syn aggregation after immunodepleting ApoA1 and ApoE. Finally, we observed that CSF ApoA1 and ApoE levels significantly correlated with SAA kinetic parameters in *n* = 31 SAA-negative control CSF samples spiked with preformed α-syn aggregates.

**Conclusions:**

Our results describe a novel interaction between lipoproteins and α-syn aggregates that inhibits the formation of α-syn fibrils and could have relevant implications. Indeed, the donor-specific inhibition of CSF on α-syn aggregation explains the lack of quantitative results from analysis of SAA-derived kinetic parameters to date. Furthermore, our data show that lipoproteins are the main inhibitory components of CSF, suggesting that lipoprotein concentration measurements could be incorporated into data analysis models to eliminate the confounding effects of CSF milieu on α-syn quantification efforts.

**Supplementary Information:**

The online version contains supplementary material available at 10.1186/s13024-023-00613-8.

## Main text

### Background

Parkinson’s disease (PD), dementia with Lewy bodies (DLB) and multiple system atrophy (MSA) are neurodegenerative diseases pathologically characterized by the presence of intracellular α-syn inclusions in vulnerable brain regions and are commonly referred to as synucleinopathies. Seed amplification assays (SAAs), known as protein misfolding cyclic amplification (PMCA) [1] and real-time quaking-induced conversion (RT-QuIC) [2] in the prion field, have been recently adapted for detecting α-syn aggregates in human biological fluid and tissues and may significantly improve the diagnosis of synucleinopathies in the near future. SAAs are based on the amplification of minute amounts of prion-like α-syn aggregates (α-syn seeds) present in biological matrices, which propagate in vitro by recruiting added recombinant α-syn monomers through cycles of elongation and fragmentation [3, 4]. The amplification process is monitored using thioflavin-T (ThT), a fluorescent dye that binds with high affinity to the cross-β-sheet motifs of amyloid aggregates. Remarkably, SAAs have detected α-syn seeds in CSF from prodromal PD cases [5, 6], reaching similar sensitivity as in patients with full-blown disease [7–9]. Initial reports showed correlations between the speed of aggregation and both disease progression (H&Y score) [1] and levels of synthetic α-syn aggregates spiked in CSF [1, 7]. However, these results were not replicated when analysing larger cohorts, [9, 10] and semi-quantification by serial dilutions did not correlate with disease progression either [7, 9]. It has been observed that CSF from both healthy controls (HC) and synucleinopathy cases inhibits α-syn aggregation compared with the sole buffer [1, 11–13]. As a result, SAA protocols include CSF dilution to overcome inhibition and enable efficient amplification of α-syn seeds [2, 11]. This effect has been repeatedly observed but, surprisingly, not yet characterized. Indeed, the effects of CSF on α-syn aggregation may explain the apparent lack of correlation between assay parameters with disease progression and α-syn burden [9, 10].

In this work, we comprehensively characterized the inhibitory effect of CSF on α-syn aggregation: we first identified a high-molecular weight (HMW) fraction of CSF exerting most of the inhibitory effect. Subsequently, we selected putative inhibitors based on their relative abundance in the CSF HMW fraction and specifically analysed their interactions with α-syn, their effect on α-syn aggregation, and possible impact on α-syn SAAs.

## Materials and methods

### Study design

**Fig. 1:**
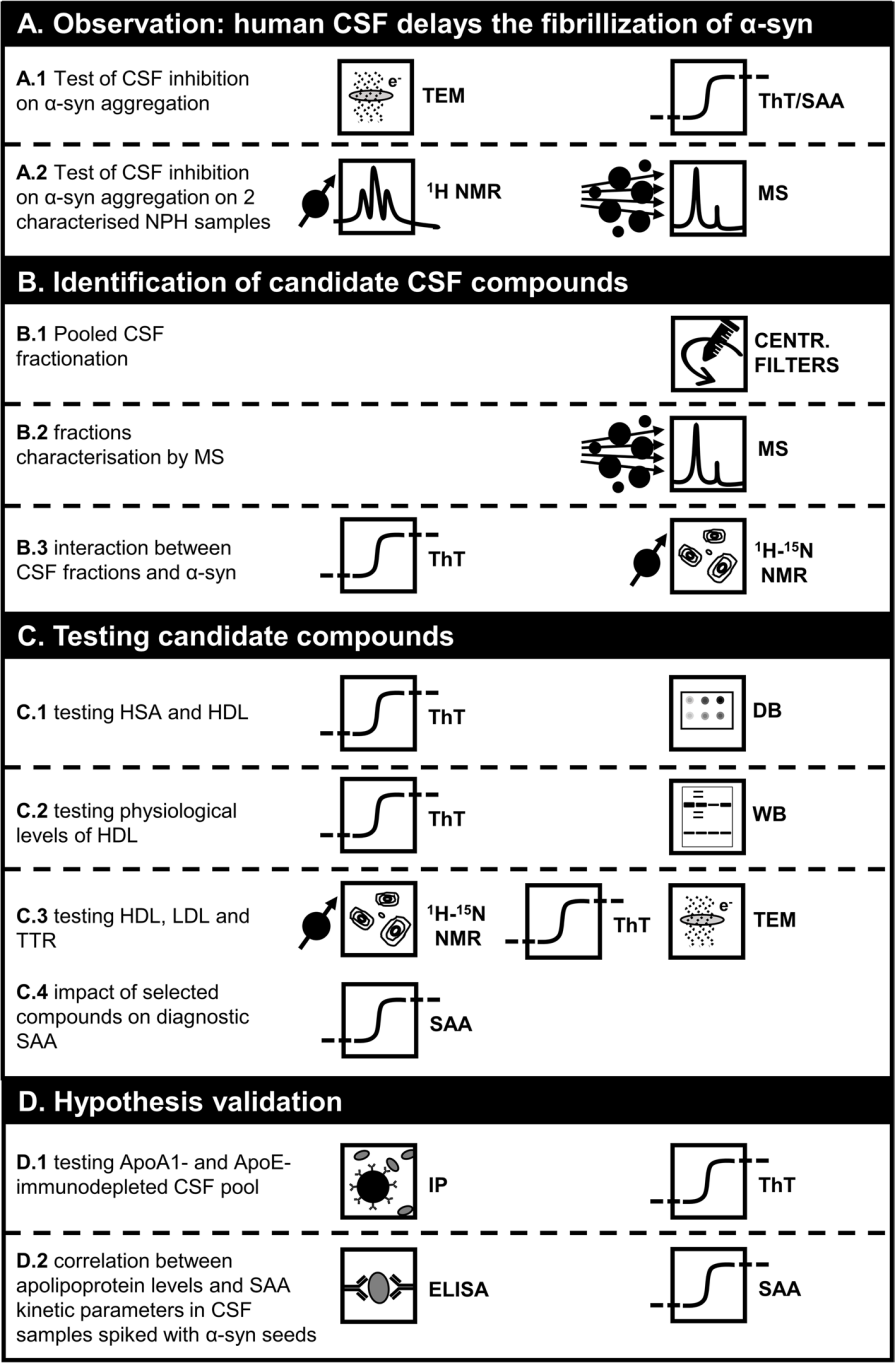
Scheme summarizing the pipeline and techniques used in the present work. Icon legend. TEM: transmission electron microscopy; ThT: thioflavin-T protein aggregation assay; SAA: α-synuclein seed amplification assay; ^1^H NMR: solution proton magnetic resonance spectroscopy; MS: mass spectrometry; CENTR. FILTERS: centrifugal filters; ^1^H-^15^N NMR: 2D proton-nitrogen solution nuclear magnetic resonance spectroscopy; DB: dot blot assay; WB: Western blot assay; IP: immunoprecipitation; ELISA: enzyme-linked immunosorbent assay

In the current work we thoroughly characterized the inhibitory effect of human CSF on α-syn aggregation by means of several complementary techniques. The study design of the present work is summarised in Fig. 1. At first, we collected more evidence supporting the fact that CSF is naturally capable of inhibiting α-syn aggregation in both SAA reaction mix and phosphate buffered saline (PBS), both in seeded and unseeded conditions, and in a patient-dependent manner (Fig. 1A.1). Starting from the observation that two CSF samples collected from normal-pressure hydrocephalus (NPH) patients with marked difference in proteins content produced different α-syn ThT fluorescence profiles in PBS (Fig. 1A.2), we performed fractionation of a pool of CSF collected from neurological controls by mean of centrifugal filters (Fig. 1B.1). We then analysed different fractions by means of mass spectrometry and Protein aggregation assays (Fig. 1B.2–3). We determined that the fraction corresponding to molecular weight above 100 kDa, rich in apolipoproteins, albumin and transthyretin (TTR), retained most of the inhibitory effect of neat CSF. By means of Protein aggregation assays, Western blot (WB), dot blot (DB), transmission electron microscopy (TEM), and solution nuclear magnetic resonance (NMR) spectroscopy we determined that high-density and low-density lipoproteins (HDL and LDL) are highly inhibitory against α-syn aggregation and likely interact with α-syn oligomeric species (Fig. 1.C.1–3). Subsequently, we tested the impact of varying (within physiological ranges) concentrations of albumin, TTR, HDL and LDL on ultrasensitive diagnostic SAA (Fig. 1C.4). We then used immunoprecipitation (IP), to deplete the two most abundant CSF apolipoproteins (ApoA1 and ApoE) from a newly made CSF pool and tested the effect with Protein aggregation assays (Fig. 1D.1). Finally, we measured total protein content, ApoA1 and ApoE concentrations in SAA-negative CSF samples of a small cohort of neurological controls and repeated SAA on the same samples spiked with preformed aggregates to evaluate possible correlation between SAA kinetic parameters and CSF total protein content, ApoA1, and ApoE concentrations (Fig. 1D.2).

### Protein expression and purification

#### Recombinant α-syn expression and purification (protein aggregation assay in PBS)

*Escherichia coli* BL21 (DE3) Gold were transformed with a pT7-7 vector cloned with the gene encoding α-syn. The overnight preculture of transformed cells was diluted 100-fold in LB medium and induced at an OD_600_ value of 0.6–0.8 with 1 mM isopropyl-β-d-thiogalactoside; after 5 h of incubation at 37 °C, the cells were harvested at 4000 rpm (JA-10, Beckman Coulter). The extraction was carried out through osmotic shock using 100 mL of the buffer Tris 30 mM, EDTA 2 mM, and sucrose 40%, at pH 7.2, according to Shevchik et al. [14] and Huang et al. [15].

The suspension was then ultra-centrifuged at 20 000 rpm (Type 70 Ti rotor, Beckman Coulter) for 25 min, and the pellet was collected and resuspended with 90 mL precooled ultrapure water containing 38 μL of 1 M MgCl_2_ and then ultra-centrifuged a second time. Supernatants derived from these two centrifugation steps were combined and dialyzed against 4 L of 20 mM Tris/HCl buffer at pH 8.0. The protein was then loaded in the fast protein liquid chromatography system, and anion-exchange chromatography was carried out with 0–50% linear gradient 1 M NaCl (GE Healthcare HiPrep Q HP 16/10 Column). The collected fractions were lyophilized and resuspended in 10 mM Tris/HCl, 1 mM EDTA, and 8 M urea at pH 8.0 for chemical denaturation. To eliminate all of the protein that formed aggregates, two size-exclusion chromatographies (HiLoad 16/600 Superdex 75 pg Column) were performed with 20 mM phosphate and 0.5 mM EDTA at pH 8.0 as the elution buffer. Purified α-syn was dialyzed against Milli-Q water and lyophilized in batches for long-term storage. The Roche complete protease inhibitor cocktail was added only during the extraction step in the quantity suggested by the producer.

^15^N-labeled wild-type α-syn was expressed in Escherichia coli grown in M9 minimal medium supplemented with ^15^NH_4_Cl and purified as started for E. coli in LB medium.

For ^15^N-labelled and unlabelled α-syn, protein expression and purification were performed as previously described [16]. 1H solution NMR, gel electrophoresis, and silver staining were used to check the quality of the purified protein. An image of two replicate silver staining experiments performed on the purified α-syn after one and two (used in Protein aggregation assays) size-exclusion chromatography steps is shown in Fig. S1 (Supplementary Material).

#### Recombinant transthyretin (TTR) expression and purification

For TTR, Escherichia coli BL21(DE3) RIPL PLysS cells were transformed with pET-28a( +) plasmid encoding TTR gene. The cells were cultured in LB Medium containing 0.1 mg/mL of Kanamycin, grown at 37 °C, until OD_600_ reached 0.6–0.8, then induced with 1 mM isopropyl β-D-1-thiogalactopyranoside. They were further grown at 37 °C overnight and then harvested by centrifugation at 6500 rpm (JA-10 Beckman Coulter) for 15 min at 4 °C. The pellet was suspended in 20 mM Tris–HCl, pH 8.5 (60 mL per litre of culture) and sonicated at 4 °C for 40 min. The suspension was centrifuged at 40,000 rpm (F15-6 × 100y Thermo Scientific) for 40 min and the pellet discarded. TTR was purified by anionic-exchange chromatography using a HiPrep Q FF 16/10 column (GE Healthcare Life Science). The protein was eluted in 20 mM Tris–HCl buffer at pH 8.6 with a linear 0–1 M NaCl gradient. Fractions containing pure TTR were identified by Coomassie staining SDS-PAGE gels, then joined and purified by Size Exclusion Chromatography using HiLoad Superdex 26/60 75 pg in 50 mM phosphate buffer at pH 7.5.

### Neurological control CSF samples

The neurological control (NC) CSF samples used in this work had been previously collected and stored at -80 °C according to international guidelines [17]. NC were cognitively unimpaired subjects referring to the Centre for Memory Disturbances of the University of Perugia (Perugia, Italy), who underwent lumbar puncture for subjective memory complaints not confirmed by the neuropsychological assessment, or as part of a diagnostic work-up for minor neurological symptoms (i.e., headache, peripheral neuropathy, psychiatric disorders), showing no cognitive impairment after at least a 2-year follow-up. All the selected NC samples tested negative for both classical AD CSF biomarkers (amyloid 1–40 and 1–42 peptides ratio, total tau, and T181-phosphorylated tau) [18] and for α-syn SAA [19].

#### CSF samples used in fractionation experiments

CSF from 19 different NC subjects (10 females and 9 males, average age = 70 y, standard deviation = 8 y) were pooled (CSF pool 1) reaching a total volume of 8 mL that was split in 2 aliquots of 3 mL and 10 aliquots of 0.2 mL.

#### CSF samples used for immunodepletion experiments

A second CSF pool of 5 mL was prepared from different CSF samples collected form *n* = 10 NC subjects (5 females and 5 males, average age = 69 y, standard deviation = 5 y). This second pool was then split in 10 aliquots of 0.5 mL each, which were then used for ApoA1 and ApoE immunodepletion experiments and protein aggregation assays.

#### CSF samples used for ELISA and SAA experiments

Two aliquots of 0.5 mL relative to 31 CSF samples collected from NC subjects (7 females and 24 males, average age = 69 y, standard deviation = 8 y), were selected for ApoA1 and ApoE ELISAs, total protein measurement, and SAA experiments.

### Healthy control and PD CSF reference samples

**Fig. 2:**
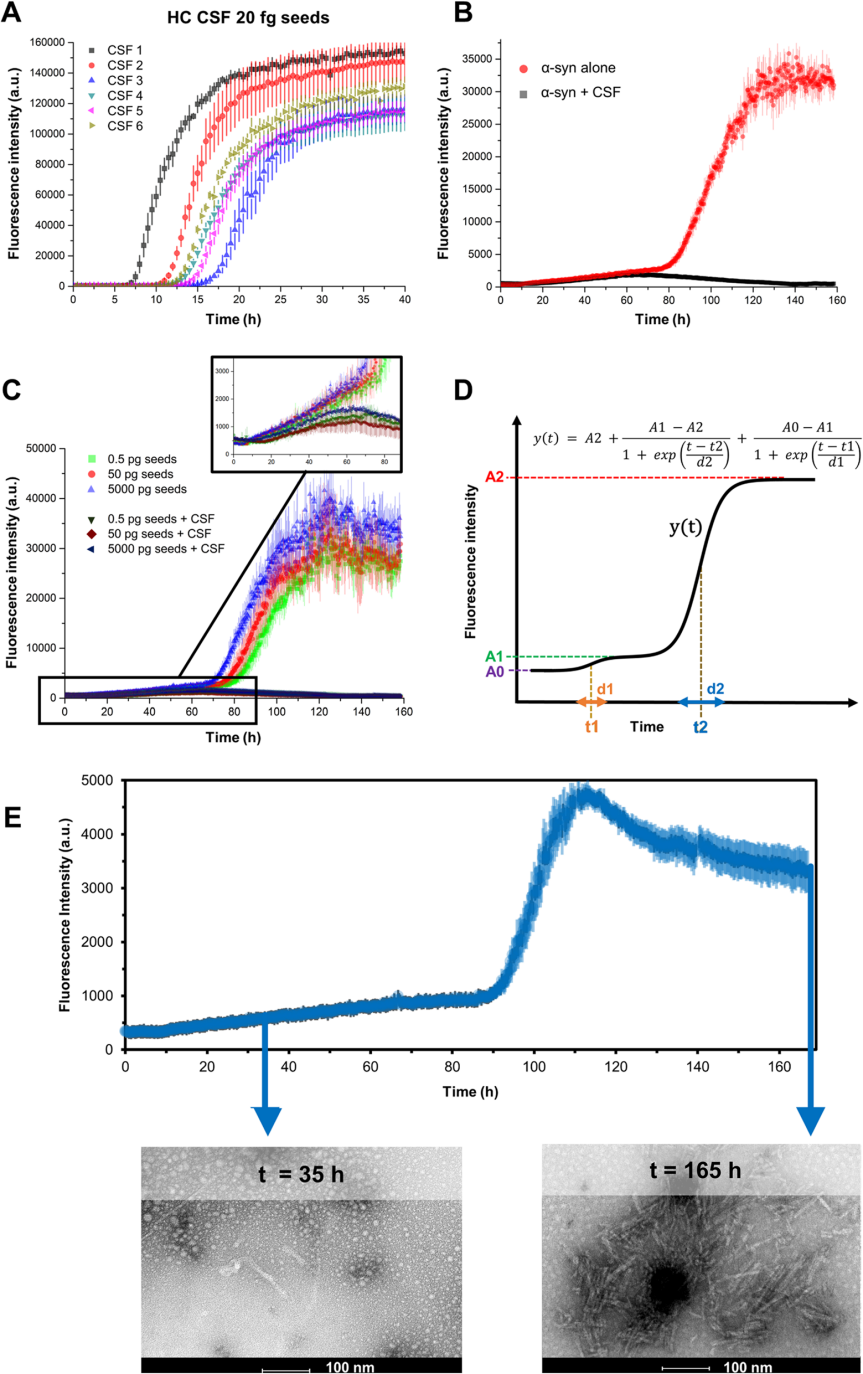
CSF inhibits α-syn aggregation in unseeded and seeded conditions in a patient-dependent manner. **A** Six different human HC CSF samples (40 µL) spiked with 20 fg synthetic preformed α-syn fibrils (seeds) and analysed by diagnostic SAAs conditions: 0.3 mg/mL (19.6 µM) of recombinant α-syn in 100 mM PIPES pH 6.5 and 500 mM NaCl, 200 µL final volume. **B** Protein aggregation assay performed using 0.7 mg/mL of recombinant α-syn in PBS with (black) and without (red) 40 μL of NC CSF pool (final volume of 200 μL). **C** Seed amplification assay in PBS of different amounts of synthetic seeds (0.5, 5, 50, 500 and 5,000 pg) with and without NC pooled CSF (only 3 seed masses shown). **D** Graphical description of the fitting function used. A2 fits the fluorescence value of the second plateau, A1 fits the fluorescence value of the first plateau and A0 fits the baseline fluorescence. The time parameters t1 and t2 fit the first and the second inflection points, respectively, while d1 and d2 represent the slopes of the sigmoids. **E** Protein aggregation assay performed using 0.7 mg/mL of recombinant α-syn in PBS (final volume of 200 μl). Six glass beads with a diameter of 1 mm were added in each well. The shaking/incubation protocol consisted in 1 min shaking at 500 rpm and 14 min rest at 37 °C. The experiment was performed in quintuplicate; three replicates were used to produce the above reported average aggregation profile, the other two replicates were collected from the plate at t = 35 h and t = 165 h, and analysed by TEM to produce the representative images shown in the bottom of panel E). All ThT fluorescence traces are represented as average intensity over 3 replicates with error bars representing SEM

CSF collected from healthy control (HC) and PD subjects was used to perform initial α-syn seed spiking experiments and to test the impact of adding HDL, LDL, HSA, and TTR at Amprion Inc. (San Diego, CA, U.S.). The HC1-6 samples used in α-syn seed spiking experiments shown in Fig. 2A have been purchased from *Biochemed Services* (Winchester, VA, U.S.) and were collected from 3 male and 3 female subjects (age = 26–39 y). With reference to the experiments summarised in Fig. 8, HC22 (female, age = 35 y) and HC24, HC66, HC67 (all males, age = 30–35 y) were also purchased from *Biochemed Services*. All neat HC CSF samples tested negative in α-syn SAA. With reference to the same experiments, PD10, PD34, PD62 CSF samples (all males, age = 72–79) belonged to PD patients with an Hoehn and Yahr (H&Y) stage of 2 and were purchased from *PrecisionMed (Carlsbad, CA, U.S.).* PD47 (PD patient, female, age = 62 y, H&Y = 1.5) was instead purchased from *BioIVT (Westbury, NY, U.S.).* All neat PD CSF samples tested positive in α-syn SAA.

### CSF Fractionation procedure

An aliquot of 3 mL of CSF pool 1 was resuspended in 1.5 mL of PBS 3 × in order to have 4.5 mL of human pooled CSF in PBS 1x. This volume was then subjected to a series of filtrations using Amicon® Ultra-4 molecular weight cut-off (MWCO) filters. The procedure used to fractionate human CSF is schematized in Fig. 4A. The aliquots collected in this way contained the different constituents of the starting 4.5 mL of CSF in PBS with different concentration factors, the volume and the relative concentration factors (with respect to fraction 1) of the aliquots depicted in Fig. 4A are summarised in Table S1. Aliquots 2, 3, 4 and 5 were washed 3 times with PBS before storage. The ability to interact with α-syn monomers was then tested for all the CSF fractions. The different concentration factors were adjusted by diluting the samples with PBS (see Supplementary Material Table S1).

### Generation of preformed α-syn aggregates

Preformed α-syn aggregates were generated by incubating 1 mg/mL of α-syn in PBS for one week at 37 °C under vigorous double orbital shaking (500 rpm) in a sealed 1.5 mL polypropylene vial. The final products were subjected to cycles of sonication (20 s tip sonication, 20 s rest) with an amplitude of 12 μm. The polypropylene vial had been immersed in ice for the whole duration of the sonication procedure. The aggregates were then diluted at 0.25, 2.5, 25, 250 and 2500 pg/μL, considering the initial monomer concentration as reference. The generated α-syn aggregates were then aliquoted and stored at -80 °C.

### α-Synuclein Seed Amplification Assay (αS-SAA)

Samples were analysed as previously reported [19, 20]. Briefly, CSF samples were evaluated in triplicate (40µL/well) in a 96-well plate (COSTAR 96, cat# 3916), in a reaction mix consisting of 0.3 mg/mL recombinant α-syn (Amprion, cat# S2020), 100 mM PIPES pH 6.50 (Sigma, cat# 80,635), 500 mM NaCl (Lonza, cat# 51,202), 10 µM ThT (Sigma, cat# T3516), and a 3/32-inch BSA-blocked Si_3_N_4_ bead (Tsubaki Nakashima). Beads were blocked with 1% BSA in 100 mM PIPES pH 6.50 for 1 h, washed twice with 100 mM PIPES pH 6.50, and dried overnight. This assay was performed in a BMG FLUOstar Omega shaker/reader at 37 °C, plates were shaken for 60 s every 30 min for 150 h. The assay outcomes of the assay are positive, inconclusive, or negative, based on a probabilistic algorithm that uses maximum fluorescence and kinetic parameters [19]. With respect to other Protein aggregation assays performed in this work, the ultrasensitive diagnostic SAA used a different αSyn substrate (C-terminal histag), which was purified by standard IMAC procedures (Amprion, cat#S2020) [19, 20].

### ThT protein aggregation assays in PBS

The protein aggregation experiments used to characterize interaction between α-syn and CSF constituents were performed with a programmable BMG LABTECH ClarioStar® fluorometer in Greiner clear-bottom 96-well plates (cat# 655,906). The ThT fluorescence was read from the bottom using excitation and emission wavelengths of 450 and 480 nm, respectively. An incubation temperature of 37 °C was used for all the experiments. Slightly different gain values were used to avoid overflow of the analog-to-digital converter. In each experiment, lyophilized recombinant α-syn was thawed in 3 mM NaOH at the concentration of 3.5 mg/mL. The solution was brought to physiological pH by diluting it with concentrated PBS (4x) and distilled water. In all the experiments described, the final reaction volume was 200 μL, α-syn final concentration was 0.7 mg/mL and ThT final concentration was 10 μM. To avoid bacterial contamination, 0.1% NaN_3_ was also present in all the tested conditions. All reactions were performed at 37 °C in sealed plates. Each condition was tested at least in triplicate.

#### Testing of the inhibitory effect of CSF and CSF fractions

158 μL of the solution containing monomeric α-syn was poured in wells, each of them containing 6 glass beads of 1 mm diameter. Depending on the experiment, 40 μL of human pooled/NPH CSF, 40 μL of PBS or 40 μL of CSF fractions were added. In seeded experiments, 2 μL of PBS containing 0, 0.25, 2.5, 25, 250, and 2500 pg/μL of α-syn aggregates were added. Plates were sealed and subjected to cycles of shaking (1 min double-orbital shaking at 500 rpm, 14 min rest) inside the fluorometer.

#### Protein aggregation assays with human serum albumin (HSA) and high-density lipoproteins (HDL)

To compare the results with those of a previously published paper, the experiment was performed in the exact same way as it is described in Bellomo et al. [16]. With respect to the above-described experiments the only notable difference consisted in applying a shaking/incubation protocol of 29 min rest and 1 min shaking. In addition to HSA (Sigma Aldrich, A1653), wells containing 0.12 mg/mL and 0.57 mg/mL human serum HDL (Sigma Aldrich, LP3) with and without α-syn were also analysed.

#### Protein aggregation assays with HDL, low-density lipoproteins (LDL) and TTR

In these experiments 40 μL of solutions containing different dilutions (all the products were diluted with distilled H_2_O) of human serum HDL (Sigma-Aldrich LP3), human serum LDL (Sigma-Aldrich LP2), recombinant TTR were added. Each condition was replicated in 3 distinct wells in the presence and absence of α-syn. For the experiments in which we tested the effect of different concentrations of human serum HDL (0, 0.003, 0.03, 0.3 and 1 mg/mL) the plate was sealed and subjected to cycles of 1 min double-orbital shaking at 500 rpm, 14 min rest. For the experiments in which we tested the effect of different concentrations of human serum HDL (0.3 mg/mL), LDL (0.03 and 0.3 mg/mL) and TTR (1.0, 0.3 and 0.03 mg/mL) the shaking protocol was changed to 2 min double-orbital shaking at 500 rpm and 13 min rest at 37 °C.

### Analysis of ThT fluorescence profiles

#### Probabilistic algorithm for α-syn SAAs

During the ultrasensitive SAA experiments performed at Amprion Inc., fluorescence readings were collected every 30 min to estimate kinetic parameters with high accuracy. The following 4 parameter sigmoid was used to fit the raw fluorescence readings:

$$\mathrm{F}\left(\mathrm{t}\right)={\mathrm{F}}_{\mathrm{min}}+\frac{{\mathrm{F}}_{\mathrm{max}}-{\mathrm{F}}_{\mathrm{min}}}{1+{\left(\frac{{\mathrm{T}}_{50}}{\mathrm{t}}\right)}^{\mathrm{S}}}$$

where *F*_*min*_ is the minimum fluorescence, *F*_*max*_ is the maximum fluorescence, *T*_*50*_ is the time to reach 50% Fmax, *S* is the slope, and *t* is time. The coefficient of determination (R^2^) was calculated for each fitting. The α-syn SAA result of each CSF sample was determined using a probabilistic algorithm:

$${\mathrm{P}}_{\mathrm{pos}}=\frac{{\mathrm{e}}^{\mathrm{A}+\mathrm{B}*{\mathrm{Fmax}}_{5000}+\mathrm{C}*{\mathrm{Rsquare}}_{93}}}{1+{\mathrm{e}}^{\mathrm{A}+\mathrm{B}*{\mathrm{Fmax}}_{5000}+\mathrm{C}*{\mathrm{Rsquare}}_{93}}}$$

where *P*_*pos*_ is the probability for a replicate to be positive, A = -4.02, B = 2.98, C = 1.87, and *Fmax*_*5000*_ and *Rsquare*_*93*_ are binary values depending on a threshold. If the *F*_*max*_ of a given replicate is above 5,000 RFU, then *Fmax*_*5000*_ = 1, else 0. If the R^2^ for the fitting of the 4-parameter model to the fluorescence data is above 0.93, then *Rsquare*_*93*_ = 1, else 0. The coefficients A, B, and C were estimated using a database of more than 900 aggregation curves. Among the PD and HC samples in the database, *Fmax* and the R^2^ showed statistically significant differences (*p*-value < 0.001 for each of these variables). If the probability for positivity (*P*_*pos*_) is higher than 0.12, the replicate is determined positive, otherwise it is determined negative. Since CSF samples were analysed in triplicates, if all 3 replicates were positive the CSF sample was called positive. If none or one replicate was positive the CSF sample was called negative. If two replicates were positive and the average *F*_*max*_ of the 3 replicates was less than 5,000 RFU (or a.u.) or the coefficient of variation (CV) was higher than 110, the sample was also called negative.

### Double sigmoid fitting

The average background fluorescence produced by three wells containing the analyte without α-syn was subtracted prior to the analysis from the ThT intensity profiles relative to the same analyte in the presence of α-syn. While analysing data relative to the sole α-syn the background fluorescence from well containing only the reaction buffer was subtracted. Each ThT kinetic trace was then fitted with a double sigmoid function using Origin Pro v9.0. In the fitting model, A2 fits the fluorescence value of the second plateau, A1 fits the fluorescence value of the first plateau and A0 fits the baseline fluorescence. The time parameters t1 and t2 fit the first and the second inflection points, respectively, while d1 and d2 represent the slopes of the sigmoids. In the non-linear fitting procedure used, the following bounds were applied: 0 < A0 < 1000, 500 < A1 < 5000, A2 > 2000, 0 < t1 < 100 h and t2 > 0. For some kinetic traces, a decrease in fluorescence was observed after reaching the second plateau. This known phenomenon is caused by the sequestration of ThT molecules by mature fibrils and by the sedimentation of HMW insoluble aggregates [21]. In these cases, the last descending part of the ThT profile was removed prior to fitting. Fitting was rejected when the adjusted determination coefficient R^2^ was below 0.3.

### WB and dot-blot assays

Equal amounts of assay products (volumes containing 2 µg of α-syn) were added with Laemmli’s sample buffer without sodium dodecyl sulphate (SDS), without boiling them to prevent solubilization of SDS-sensitive aggregates. Samples were separated through SDS-PAGE on 4–20% polyacrylamide gels (Bio-Rad) and transferred into PVDF membranes (0.45 μm, Bio-Rad) by wet transfer at 100 V constant for 90 min using 25 mM Tris–HCl, 192 mM glycine, 20% methanol, and 0.015% SDS. Membranes were fixed with 4% PFA for 30 min prior to blocking with 5% non-fat milk in TBS-T (TBS with 0.1% Tween 20) for 1 h at room temperature. After blocking, filters were incubated with primary antibody against α-syn (211, sc-12767, Santa Cruz Biotechnology) O/N at 4 °C. The membranes were further incubated with a goat-anti-mouse IgG-HRP conjugate (Bio-Rad, 1,706,516; 1:5000) secondary antibody for 1 h at RT, and signals were visualized using an ECL reaction. Densitometric analysis was performed with ImageJ software (National Institute of Health). Dot-blot images were inverted, and window/level adjusted prior to the analysis. A rectangular region of interest (ROI) was used to measure the average grey level intensity in each band relative to monomeric α-syn. The average grey level of an equivalent ROI not containing bands was subsequently subtracted. To estimate the approximate percentage of remaining α-syn monomer, the average intensity of the WB bands was divided by the one at t = 1 h.

For dot blotting aliquots of products obtained in SAAs corresponding to 300 ng of monomeric α-syn in the initial reaction mixtures were spotted (2 μL/spot) on nitrocellulose membrane pre-equilibrated with TBS-T. Samples were dried at RT and fixed with PFA (0.4% in PBS) for 30 min, and then filters were blocked with 2% non-fat milk (in TBS-T). The membranes were incubated overnight at 4 °C with OC (1:1000) or A11 (1:1000) conformational antibodies [22] followed by incubation with goat-anti-rabbit IgG-HRP conjugate (1:5000; 1,706,515 Bio-Rad) secondary antibody for 1 h at RT. Membranes were developed with an ECL reaction and images were acquired through a ChemiDoc™ imaging system. Dot-blot images were inverted, and window/level adjusted prior to the analysis. The average grey level intensity was extracted from a circular ROI containing all the α-syn containing samples. For each dot, the average grey level intensity of an adjacent ROI not containing any dot was subtracted to remove the background intensity. The averaged and background-subtracted grey level intensity was then multiplied for the ROI area in cm^2^ to estimate the integrated density. No significant grey level fluctuations were found while repeating the same procedure for control samples not containing α-syn.

### Depletion of CSF ApoA1 and ApoE by immunoprecipitation

Immunoprecipitation (IP) was performed to deplete ApoA1 and ApoE from CSF pool sample. 50 µL of settled Immobilized Protein A/G (100 µL resin slurry, Pierce™ Protein A/G Plus Agarose, ThermoFisher Scientific™, USA) and 60 µg of anti-ApoA1 antibody (MIA1404, ThermoFisher Scientific™, USA) were combined in a 2-mL tube. The beads-antibody slurry was incubated for 4 h at 4 °C (constant rotation). The tube was centrifuged at 1,000 × g for 2 min at 4 °C and the supernatant was discarded. The bead pellet was washed with Phosphate Buffered Saline (PBS) twice. Similarly, 50 µL of settled Immobilized Protein A/G (100 µL resin slurry, Pierce™ Protein A/G Plus Agarose, ThermoFisher Scientific™, USA) and 20 µg of anti-ApoE antibody (PA5-27,088, ThermoFisher Scientific™, USA) were combined a 2-mL tube. The beads-antibody slurry was incubated for 4 h at 4 °C (constant rotation). The tube was centrifuged at 1,000 × g for 2 min at 4 °C and the supernatant was discarded. The bead pellet was washed with PBS twice. Subsequently, ApoA1-antibody-bound beads and ApoE-antibody-bound beads were combined in a single 2-mL tube. The solution was gently mixed and centrifuged at 1000 × g for 2 min at 4 °C. The supernatant was discarded and 400 µL of CSF pool was added to the tube with beads. The sample underwent overnight incubation at 4 °C (constant rotation). At the end of incubation, the CSF-beads slurry was centrifuged (1,000 × g for 2 min at 4 °C) and the supernatant and bead pellet were collected separately for further analysis. In parallel, a control sample was prepared by following the same procedure on beads without anti-ApoA1 or anti-ApoE antibody bound to IP. Briefly, 50 µL of neat resin slurry was transferred to 2-mL tube and washed with PBS twice. After the second wash cycle, PBS was removed and 400 µL of CSF pool was added instead. The sample underwent overnight incubation at 4 °C (constant rotation). At the end of incubation, the sample was centrifuged (1,000 × g for 2 min at 4 °C) and the supernatant and bead pellet were collected separately for further analysis. Performance of ApoA1 and ApoE IP was assessed by Western Blot. Briefly, all the samples (1. IP CSF pool sample, 2. CSF pool sample undergoing procedure mimicking IP (without antibody), 3. bead pellet of IP CSF pool sample, 4. bead pellet of CSF pool sample undergoing procedure mimicking IP (without antibody), 5. neat CSF pool sample which did not undergo any treatment) were resuspended in a reducing loading buffer and boiled 5 min at 95 °C (thermoblock). Tubes with bead pellet (samples 3. and 4.) were centrifuged and the supernatant was collected and used for further analysis. A 12% polyacrylamide gel was prepared, and the samples were run in reducing conditions. All samples were transferred from the gel to a nitrocellulose blotting membrane (SF110B, Himedia, India). The quality of the transfer was evaluated by the reversible Ponceau S staining. The membrane was blocked and subsequently probed overnight at 4 °C (shaker) with the selected primary antibodies (MIA1404 for ApoA1, PA5-27,088 for ApoE, 1:1000) diluted in 5% bovine serum albumin (BSA), 0.02% NaN_3_ in Tris-buffered saline with 0.1% Tween®20 (Sigma-Aldrich, USA) (TBST) with addition of a phenol red as a pH indicator. After the incubation, the membrane was washed with TBST and the secondary antibodies diluted 1:5000 in a blocking buffer were applied for 1 h at RT. Depending on the used primary antibodies, goat anti-mouse (170–6516, Bio-Rad, USA) (for MIA1404) or goat anti-rabbit IgG-HRP (170–6515, Bio-Rad, USA) (for PA5-27,088) were added. Signal development was performed with use of the enhanced chemiluminescence (ECL) solution (SuperSignal™ West Pico Plus, ThermoFisher Scientific™, USA).

### ApoA1 and ApoE levels in CSF

CSF levels of ApoA1 and ApoE were assessed with use of the commercially available ELISA kits—Human Apolipoprotein AI ELISA Kit, ab108803 and Human Apolipoprotein E ELISA Kit, ab108813 (Abcam, UK) in *n* = 31 SAA-negative NC CSF samples. Both assays were performed according to the manufacturer protocol. All the standard curve points and CSF samples were run in duplicate. Optical density (OD) was read at 450 nm (wavelength correction 570 nm) by the Clariostar (BMG Labtech, Germany) plate reader. The 4-parameter logistic model was applied to generate a standard curve and interpolate concentrations of analysed CSF samples.

### Total protein content of CSF samples

Total protein content was evaluated in the same samples with use of the Pierce™ 660 nm Protein Assay Reagent cat. 22,660 (Thermo Scientific™, USA). The assay was performed according to the manufacturer protocol. A set of BSA dilutions of known concentrations served as the standard to calculate total protein content of CSF samples. All the standard curve points and CSF samples were run in duplicate.

### Statistical analysis

One-way analysis of variance (ANOVA) and Tukey post-hoc test for mean comparisons were applied while assessing differences among fitted kinetic parameters for different samples. Correlation between added seed mass and T2 parameters were computed according to Spearman. Two-tailed Student’s t-test was applied when comparing adjusted integrated densities of dot blot images. Standard error of mean (SEM) is reported in each image showing bar plots and/or average fluorescence profiles. In the analysis of mass spectrometry data, an false discovery rate (FDR) of 1% was imposed and the criterion used to accept protein identification included probabilistic score sorted by the software. Correlations among SAA parameters and Log2-transformed CSF levels of ApoA1, ApoE and total protein were computed by means of Pearson’s correlation coefficients. Ward linkage criterion was applied for hierarchical clustering of correlations.

## Results

A detailed description of the study design (experiments type and workflow) is present in Sect. 2.1 of Material and Methods and graphically summarised in Fig. 1.

### Human CSF delays the fibrillization of α-syn

CSF inhibition of α-syn aggregation in SAAs remains poorly characterized, although it has been already reported in the literature [1, 2, 11, 12]. We spiked 20 fg of synthetic α-syn aggregates (synthetic seeds) in CSF of healthy control subjects (HC) to determine if the CSF inhibition is similar and intrinsic to all CSFs or if it is subject dependent. These spiked samples were analysed in a highly sensitive and specific diagnostic α-syn SAA developed by Concha-Marambio et al*.* [19, 20]. Although synthetic seeds are known to aggregate in a very reproducible way when spiked into buffer only, the kinetic of aggregation was visibly different depending on the CSF donor (Fig. 2A). Under these experimental conditions, seeded α-syn aggregation presents a single elongation phase and plateau, with the time to reach 50% of the fluorescence plateau (T50) describing the speed of the reaction. T50 values were significantly different among different CSF samples (*p* <  < 0.001, see Supplementary material Fig. S2). The least inhibitory CSF (#1) allowed for amplification of the synthetic seeds ~ 50% faster than the most inhibitory CSF (#3) (Supplementary Material Fig. S2). CSF samples without synthetic seeds did not show any spontaneous aggregation under this protocol. We then decided to further characterize this inhibition by ThT fluorescence in a simplified protein aggregation assay able to better highlight the inhibitory effect of CSF, even on α-syn spontaneous aggregation, in a buffer with physiological pH and ionic strength (PBS). A pool of CSF samples collected from neurological controls (NC) was added to the α-syn/PBS reaction mixture in the absence of synthetic seeds to evaluate if CSF inhibition could suppress α-syn spontaneous aggregation. In these experimental conditions, CSF completely blocked the spontaneous aggregation of α-syn (Fig. 2B). We then compared CSF inhibition to seed amplification reactions in PBS buffer when adding different levels of synthetic seeds (0.5 pg – 5 ng) and found that CSF impeded the seeded aggregation of α-syn even when using high concentrations of synthetic α-syn seeds (5 ng), while reactions without CSF enabled reproducible seeded aggregation in all tested concentrations (Fig. 2C). Interestingly, in these physical–chemical conditions, both seeded and spontaneous aggregation presented two elongation phases and two plateaus (Fig. 2C, inset), which could be described by a double-sigmoidal function (Fig. 2D). The inflection point of the second elongation phase (t2) was estimated using a double-sigmoidal fitting and correlated with the log mass of synthetic seeds (Supplementary MaterialFig. S3), in agreement with previous literature [1, 12]. To further characterize our experimental conditions in PBS, we analysed samples from both plateaus of the aggregation curve by TEM. In the first plateau, only short and partially amorphous aggregates are present, whereas a high number of fibrillary structures is observed at the second plateau (Fig. 2E).

**Fig. 3:**
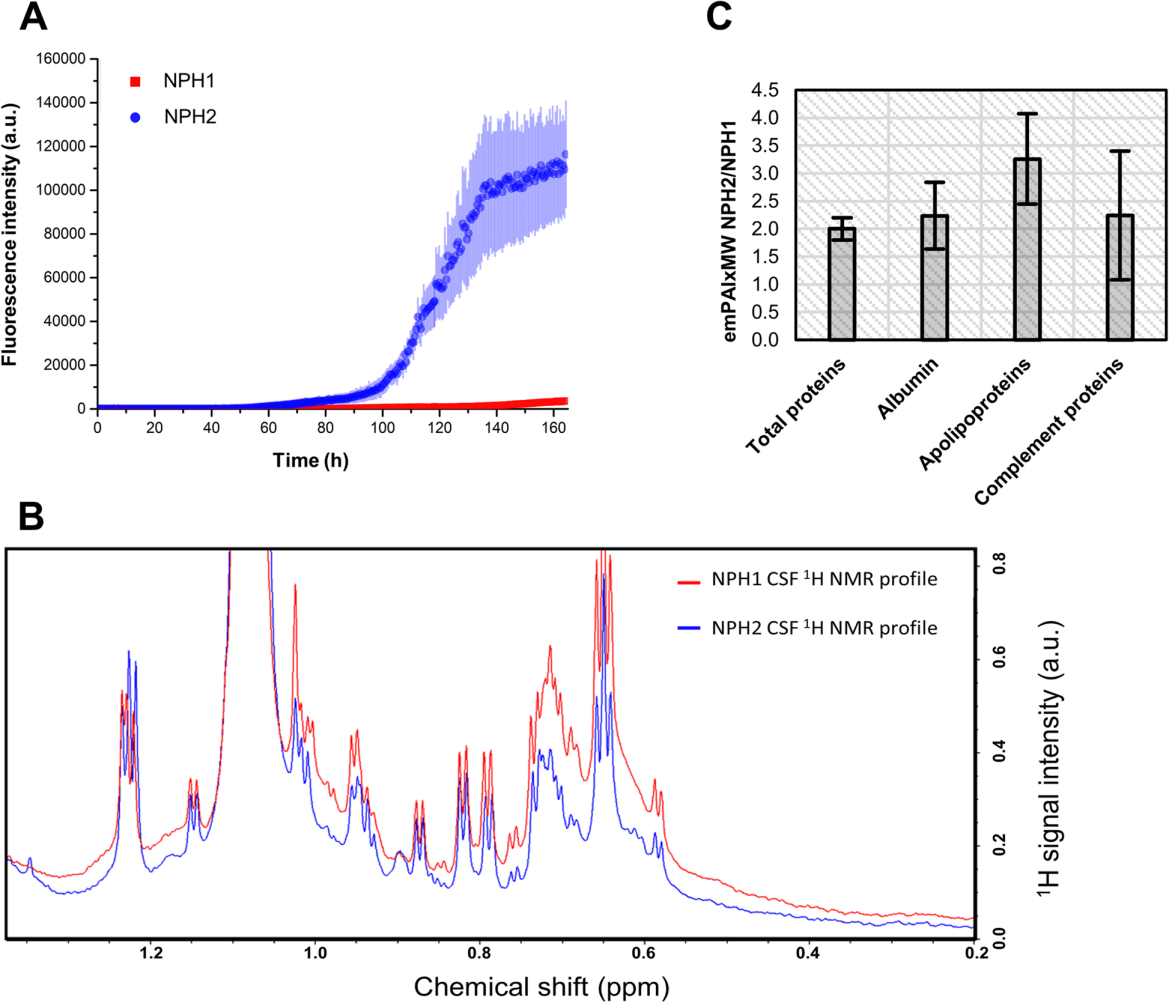
Analysis of NPH CSF samples. **A** Protein aggregation assay performed using 0.7 mg/mL of recombinant α-syn in PBS with 40 μl of CSF from 2 NPH subjects (40 µL). The two ThT fluorescence traces are represented as average intensity over 3 replicates with error bars representing the SEM. **B** Portion of 1D ^1^H NMR spectra relative to the two NPH CSF samples. **C** Relative concentration (emPAI score multiplied by protein molecular weight) ratio of total protein and the three most abundant protein constituents measured by nLC-nESI HRMS/MS in neat NPH1 and NPH2 CSF samples. Approximately 200 and 400 different proteins were detected in NPH1 and NPH2, respectively. Albumin was the most abundant protein followed by apolipoproteins and complement proteins. Apolipoproteins scores were summed together, with ApoA1 and ApoE being the most abundant (~ 85% of the total). Complement C3 and C4 were found as the most abundant complement proteins (~ 65% of the total)

CSF from cognitively unimpaired normal-pressure hydrocephalous (NPH) is usually available in large volumes and is often used for assay development or as negative controls in diagnostic SAAs. Although considered negative controls due to lack of detectable α-syn seeds, dilution of CSF components has been reported to be common in NPH patients [23] which could have relevant effects on α-syn aggregation. Thus, we evaluated the effect of CSF from 2 NPH cases on α-syn spontaneous aggregation, to determine if they replicated the inhibitory effect observed with NC-CSF (Fig. 3A). Strikingly, α-syn spontaneous aggregation was observed with the NPH2 sample, but not with NPH1, dramatically confirming that the inhibitory effect of CSF on α-syn aggregation is donor-dependent. Moreover, given the dilution effect described for some NPH patients, these results indicate a concentration-dependent inhibitory effect of some CSF components. To qualitatively test this hypothesis, we analysed the content of these two CSF samples by 1D ^1^H solution high-field nuclear magnetic resonance (NMR) spectroscopy. The most apparent differences between the two spectra lay between 1.5 and 0 ppm, where methyl resonances are usually observed (Fig. 3B). Interestingly, some species appear to be more concentrated in the CSF of NPH1 with respect to NPH2, following the pattern of α-syn aggregation inhibition. From metabolomics studies, fatty acids-rich lipoproteins and/or other HMW proteins are known to produce similar broad peaks in ^1^H solution NMR spectra [24, 25]. The qualitative information obtained by ^1^H NMR was then confirmed by nLC-nESI HRMS/MS. We identified approximately 400 and 200 different proteins in NPH1 and NPH2, respectively. Albumin, apolipoproteins and complement proteins were the three most abundant protein families in neat NPH1 and NPH2 CSF samples. In NPH1 CSF, total protein content was lower by a factor of 2 (a factor of 3 for total apolipoproteins) with respect to NPH2 CSF (see Fig. 3C).

### Fibrillization of α-syn is inhibited by high-molecular weight constituents of CSF

**Fig. 4:**
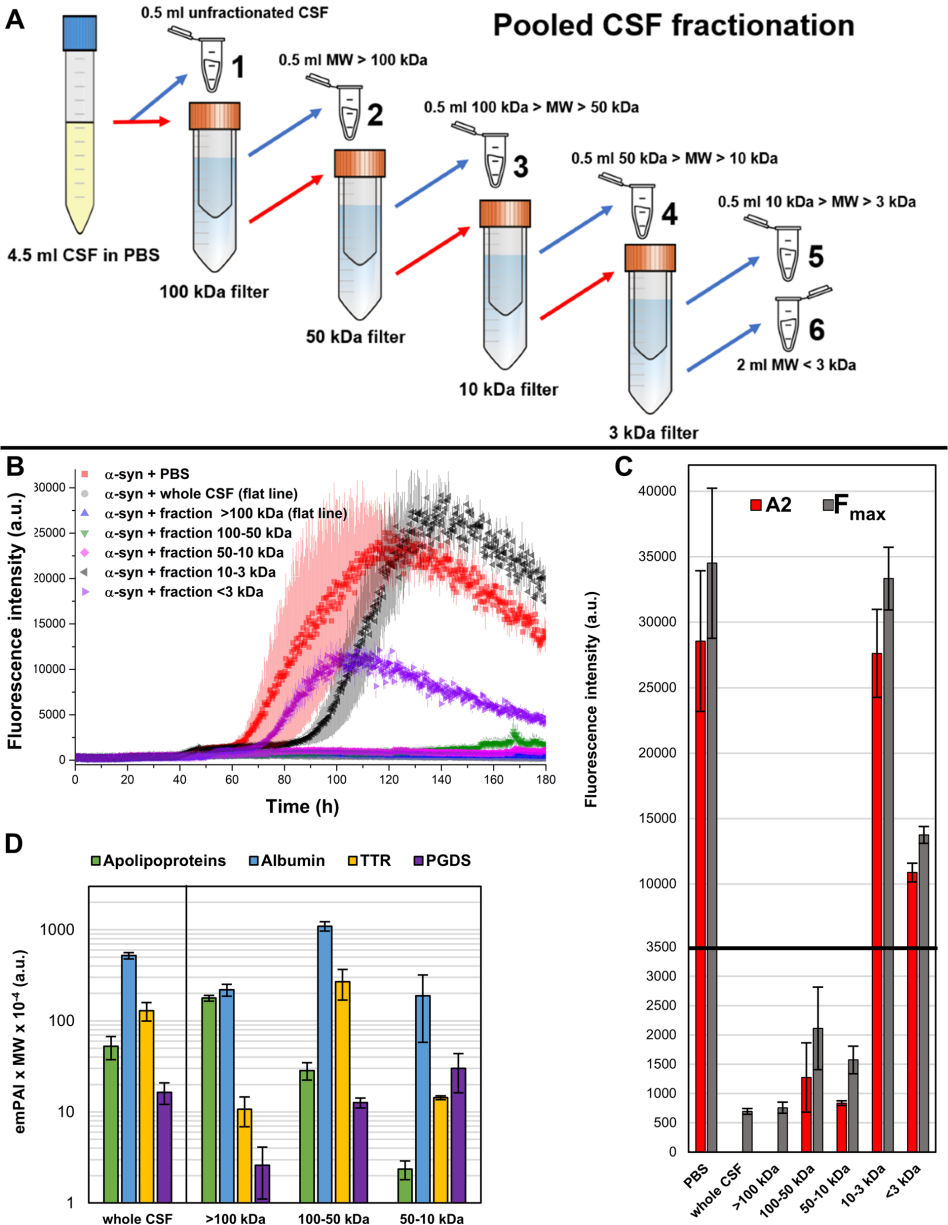
Different CSF fractions differently affect α-syn aggregation.** A** Scheme of the CSF fractionation procedure. From a starting aliquot of 4.5 mL of CSF in PBS 1x, we collected 6 aliquots containing compounds of different molecular weight and froze them in liquid nitrogen. After every filtration with centrifugal filters, the flow-through of the filtered fraction was passed to a filter with smaller cut-off.** B** The addition of CSF fractions, whole NC-CSF pool, and PBS (40 μL) was analysed by ThT protein aggregation assay to evaluate effects on α-syn spontaneous aggregation. Background signal was corrected by subtracting the average fluorescence of three replicates containing PBS, whole CSF and CSF fractions without α-syn. All ThT fluorescence traces are represented as average intensity over 3 replicates with error bars representing the SEM. **C** Mean fitted A2 parameters (fitting was not possible for samples with whole CSF and the > 100 kDa fraction) and maximum fluorescence values (F_max_) estimated from individual ThT traces. Two scales of fluorescence intensity were used to better compare the results. Represented values correspond to the average of three replicates with error bars reflecting the SEM. **D** Relative concentration (emPAI score multiplied by protein molecular weight) of the most abundant protein constituents measured by nLC-nESI HRMS/MS. Apolipoproteins scores were summed together, with ApoA1 and ApoE being the most abundant (~ 85% of the total). Scores for fractions 10–3 and < 3 kDa are not shown since the protein content of these fractions was negligible with respect to the others

After demonstrating that CSF composition is donor-dependent and correlates with inhibition of α-syn aggregation in vitro, we fractionated the previously used NC-CSF pool to investigate which CSF components were behind this inhibitory effect. Fractionation was performed using conical centrifugal molecular weight cut-off (MWCO) filters (for details about fractionation procedure and final concentration factors see Fig. 4A and Table S1). We obtained 6 samples after fractionation: whole CSF, CSF constituents of molecular weight (MW) above 100 kDa (> 100 kDa), CSF constituents of MW between 100 and 50 kDa (100-50 kDa), CSF constituents of MW between 50 and 10 kDa (50-10 kDa), CSF constituents of MW between 10 and 3 kDa (10-3 kDa), and CSF constituents of MW below 3 kDa (< 3 kDa). We then analysed the inhibitory effect of each of these 6 fractions on the spontaneous aggregation of α-syn in the previously mentioned PBS conditions (i.e., those of Fig. 2B, C, E). There were clear differences in α-syn aggregation depending on the MW of the CSF fraction. Whole CSF and all the fractions with MW > 10 kDa drastically inhibited α-syn aggregation, while 10-3 kDa and < 3 kDa fractions showed comparable aggregation to the reaction without CSF components (PBS control) (Fig. 4B). We estimated the second fluorescence plateau (A2) using the double sigmoidal model and compared the results to the maximum fluorescence readings (F_max_) for all 6 CSF-derived samples (Fig. 4C). As expected, A2 and F_max_ were very similar within each CSF derived sample, confirming the goodness of the fit, except for whole CSF and > 100 kDa for which fitting was not possible. Indeed, for whole CSF and > 100 kDa, fluorescence readings remained practically flat, impeding the fit through the double sigmoidal model. Suggesting lower inhibition of α-syn aggregation, A2 and F_max_ were substantially higher in both 10–3 and < 3 kDa fractions, which were comparable to the PBS control. Some inhibition was observed in the < 3 kDa fraction, but this is probably the result of an increase in pH by air exposure during the fractionation procedure rather than an inhibition of α-syn aggregation by protein components (as described in Supplementary Material Solution NMR experiments on CSF fractions and Fig. S4-5). A2 and F_max_ dropped significantly for 50–10 and 100–50 kDa fractions, consistent with a higher degree of α-syn aggregation inhibition, while whole CSF and > 100 kDa were the most inhibitory (Fig. 4C). Estimations of t2 show similar results, with whole CSF and > 100 kDa significantly slowing down aggregation (Supplementary Material Fig. S6). These results indicate that the main inhibitory components of CSF are enriched in the > 100 kDa fraction, which retained the same inhibitory effect as whole CSF. To identify protein candidates responsible for the inhibition of α-syn aggregation, we analysed the CSF derived samples by nLC-nESI HRMS/MS (Fig. 4D). We could not detect relevant amounts of proteins in the 10–3 kDa and < 3 kDa fractions by nLC-nESI HRMS/MS (just very low levels of albumin or albumin fragments in the 10-3 kDa fraction, see Supplementary Material, Table S2). In whole CSF, the most abundant of the ~ 300 proteins detected were albumin, transthyretin (TTR), apolipoproteins, and prostaglandin-D synthase (PGDS, also known as β-trace protein). Albumin and PGDS were found not particularly enriched in the > 100 kDa fraction, suggesting low inhibition effect of these two proteins on α-syn aggregation. Following the inhibitory effect on α-syn aggregation, apolipoproteins were more abundant in whole CSF and the > 100 kDa fraction. ApoA1 and ApoE were the most abundant apolipoproteins in the > 100 kDa fraction (~ 80%), while ApoJ and ApoD accounted for ~ 6% each. The higher abundance of fatty acids-rich lipoproteins and/or other HMW proteins in NPH1 vs NPH2, and the high abundance of lipoproteins in the most inhibitory fraction of CSF (> 100 kDa) suggest that these compounds in CSF could be the main driver of the inhibition of seeded and spontaneous aggregation of α-syn.

### Human lipoproteins delay α-syn aggregation already at sub-physiological CSF concentrations

**Fig. 5:**
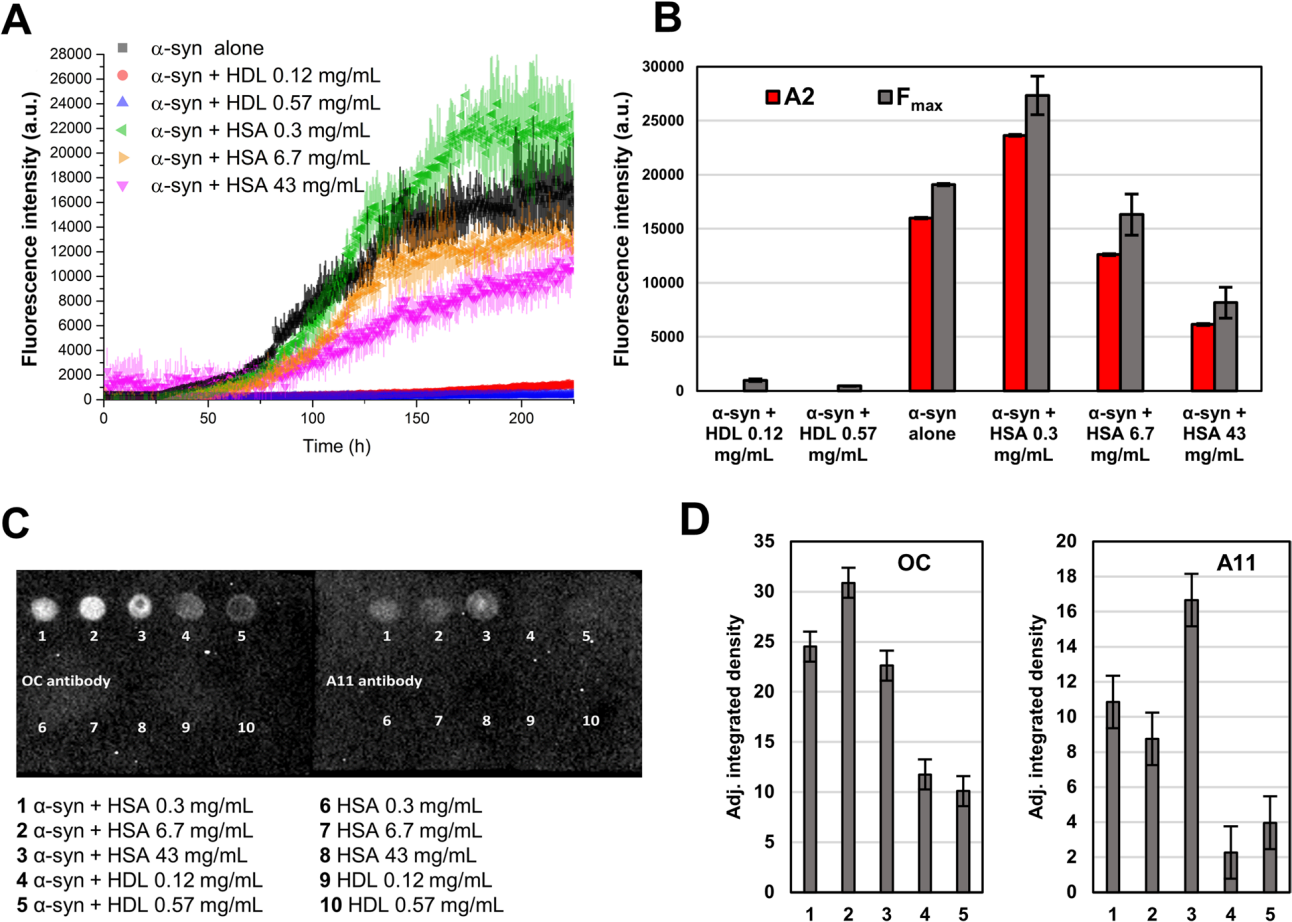
HDL reduces α-syn aggregation more efficiently than HSA. **A** ThT protein aggregation assay performed using 0.7 mg/mL of recombinant α-syn in PBS pH 7.4 in the presence of different concentrations of HSA (0, 0.3, 6.7 and 43 mg/mL) and HDL (0, 0.12 and 0.57 mg/mL). To remove the background fluorescence, the average fluorescence of three replicates containing the same amount of HSA and HDL without α-syn was subtracted prior to the analysis. The data represent the average fluorescence of three replicates with error bars representing the SEM. **B** F_max_ and fitted A2 parameters fitted from individual traces and averaged on the three replicates are shown. **C** Inverted and window/level-adjusted image of the dot-blot assay performed on the final reaction products of HSA and HDL-containing samples. Dot-blots were probed with OC (detection of fibrils) and A11 (detection of amorphous oligomers) conformational antibodies. **D** Adjusted (background subtracted) integrated density measured in a circular region of interest (0.35 cm^2^) surrounding dots relative to samples 1–5 with error bars representing the standard deviation of the background noise. The average grey level is significantly lower (*p* < 0.001) for samples with HDL with respect to all the HSA concentrations tested by applying two-tailed Student’s t-test both for OC and A11 antibodies

After identifying lipoproteins as main candidates to explain the inhibitory effect of CSF on α-syn aggregation, we evaluated if the effect is reproduced when using purified lipoprotein in the absence of the other CSF components. We used serum purified high-density lipoprotein (HDL) (LP3, Millipore-Sigma) and serum purified human serum albumin (HSA, A1653, Millipore-Sigma) as control. Albumin levels were quite similar between CSF fractions, and we have already shown that HSA only partially reduces α-syn aggregation at plasma concentrations (43 mg/mL) [16]. We tested these proteins at 0.12 and 0.57 mg/mL for HDL, and 0.3, 6.7, and 43 mg/mL for HSA (Fig. 5A). As expected, spontaneous aggregation of α-syn in sole buffer presented the double sigmoidal behaviour and reached high fluorescence values, while HSA presented marginal inhibitory effect even at the highest concentration (plasma levels). Lower concentrations did not have an inhibitory effect on α-syn aggregation. In the case of HDL, there was a striking inhibitory effect on α-syn aggregation. Moreover, this inhibition was dose-dependent since there was a minor increase in fluorescence with 0.12 mg/mL HDL and complete lack of aggregation when using 0.57 mg/mL HDL. Evaluation of F_max_ and A2 showed agreement between both parameters, except for HDL since the lack of aggregation did not allow the fitting of the double sigmoidal model (Fig. 5B). The analysis of A2/F_max_ for low HSA concentrations showed no inhibitory effect on α-syn aggregation (Fig. 5B). As complementary/orthogonal approach, we performed dot blot analysis of the reaction products using A11 and OC conformational antibodies that detect oligomeric amorphous and fibrillary aggregates, respectively [22]. As shown in the inverted and window-level adjusted image with both OC and A11 antibodies (native image and time-course of α-syn alone samples is present in Supplementary Material Fig. S7), there was formation of α-syn aggregates (samples 1, 2, and 3) in the presence of all tested HSA concentrations (Fig. 5C). However, HDL significantly reduced the amount of α-syn aggregates at both 0.12 and 0.57 mg/mL concentrations (samples 4 and 5), confirming the inhibitory effect of HDL on α-syn aggregation. We also evaluated reactions without α-syn by dot blot, to confirm that the antibodies signals were not an artifact of the serum purified proteins (samples 6–10). Quantification of the dot blot signal showed significantly lower levels of α-syn aggregates in reactions containing HDL versus HSA (*p* < 0.001, Fig. 5D).

**Fig. 6:**
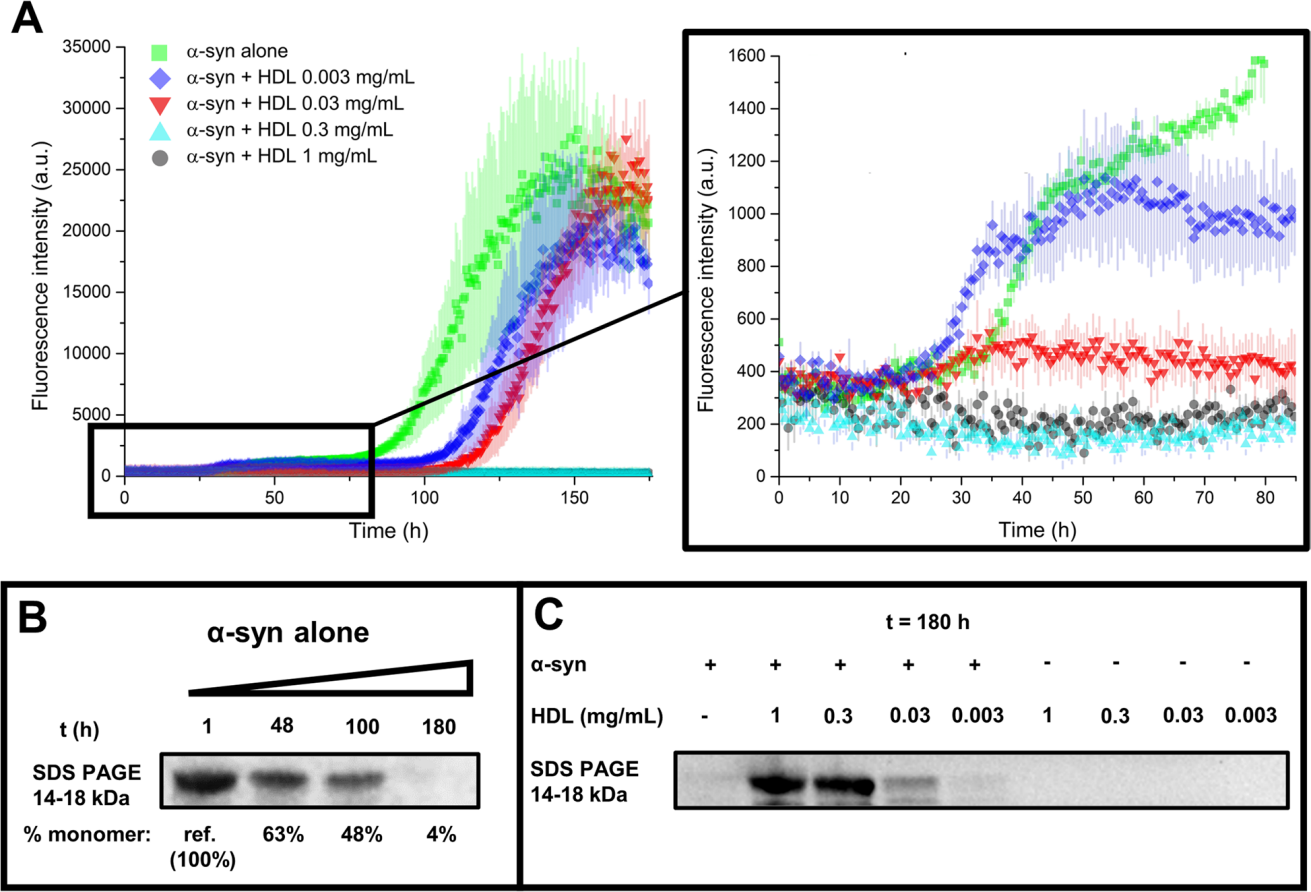
HDL reduces α-syn aggregation even at CSF physiological (ca. 0.03 mg/mL) and sub-physiological levels by preventing the formation of transient oligomeric/protofibrillary species. **A** Protein aggregation assay performed using 0.7 mg/mL of recombinant α-syn in PBS with 0, 0.003, 0.03, 0.3 and 1 mg/mL of added human serum HDL. To remove the background fluorescence, the average fluorescence of three replicates containing the same amounts of HDL without α-syn was subtracted prior to the analysis. All ThT fluorescence traces are represented as average intensity over 3 replicates with error bars representing SEM. **B** The presence of monomeric α-syn (14–18 kDa) in samples collected at different timepoints of the spontaneous aggregation process were monitored by WB using Syn211 antibody. Monomeric α-syn decreases as t increases due to the formation of fibrils. **C** In a similar way, a WB with Syn211 was performed on the reaction products obtained after 180 h, at different HDL concentrations with and without α-syn

After confirming that purified HDL at plasma concentration replicates the inhibition of α-syn aggregation shown by whole CSF and by the > 100 kDa CSF fraction, we evaluated if HDL could retain the same level of inhibition when tested within a range of concentrations from 1 to 0.003 mg/mL (including the physiological ones of human CSF). Interestingly, we observed a dose-dependent partial inhibition of α-syn aggregation when adding 0.003 and 0.03 mg/mL, while 0.3 and 1 mg/mL completely blocked the formation of ThT-reactive aggregated species (Fig. 6A). The partial inhibition was most noticeable as a delay in the second inflection point (t2), although it was also observed as a reduction in fluorescence of the first plateau (A1) (Fig. 6A inset). ApoE and ApoA1 represent 50–60% of total CSF apolipoprotein, and their respective reported concentration in CSF is approximately 0.01 mg/mL and 0.004 mg/mL [26]. Considering 0.03 mg/mL as the physiological concentration of HDL in CSF, our results show that HDL partially inhibited α-syn aggregation at a physiological concentration and at a tenfold lower concentration. Although our experiments were performed with highly purified recombinant α-syn and other components were shown not to form ThT, OC, or A11 detectable aggregates, we used WB to detect monomeric recombinant α-syn as a secondary read-out for this experiment. In agreement with ThT readings, in the absence of HDL, there was a decrease in monomeric α-syn at the time of the first plateau (48 h) and most of the monomer was consumed by the time of the second plateau (180 h) (Fig. 6B, Supplementary Material Fig. S8A). However, we detected monomeric α-syn in the presence of HDL after 180 h of the reaction, with the monomer signal being found increased at increasing concentrations of HDL (highest at 1 mg/mL HDL and the lowest in absence of HDL, Fig. 6C). However, it is worth mentioning that, in the presence of 1 mg/mL HDL, we observed an additional band (around 150–200 kDa) of much lower intensity than the monomer band (Supplementary Material Fig. S8B-C), suggesting that high concentrations of HDL may have stabilised some prefibrillar oligomers, preventing their conversion into fibrils. Collectively, these results show that purified serum HDL (at physiological concentrations and in the absence of CSF milieu) is a potent inhibitor of α-syn aggregation, comparable to whole CSF and the > 100 kDa CSF fraction.

**Fig. 7:**
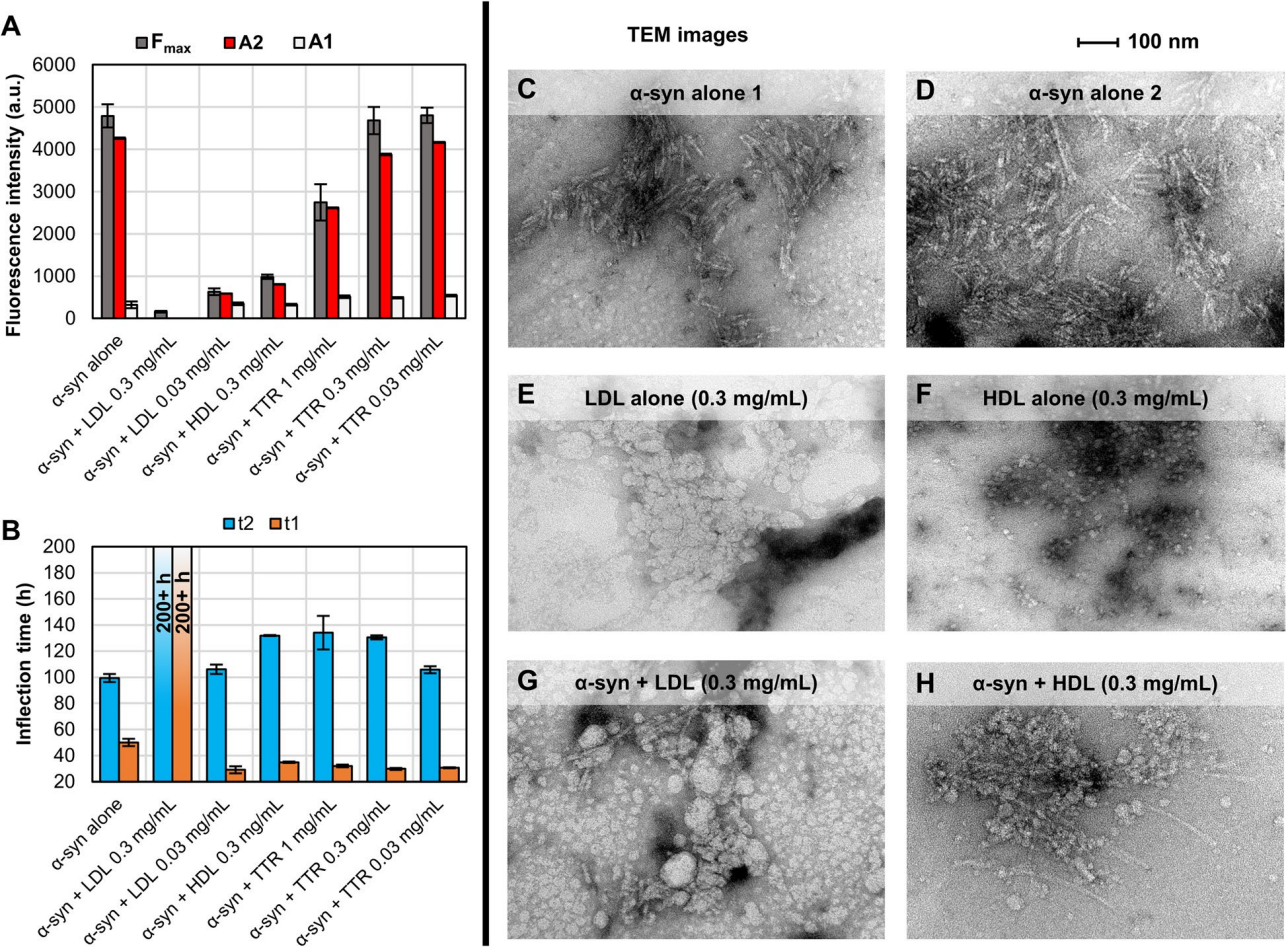
HDL and LDL impede α-syn aggregation more efficiently than TTR. Lipoproteins exert their anti-aggregation properties by interlacing to early protofibrillary/oligomeric species. **A**-**B** Fitted kinetic parameters of a Protein aggregation assay performed using 0.7 mg/mL of recombinant α-syn in PBS with different concentrations of LDL, HDL and TTR. The average fluorescence of three replicates containing the same amounts of LDL, HDL and TTR without α-syn was subtracted prior to the analysis. **C**-**H** TEM images relative to the final products obtained using (**C**-**D**) α-syn alone (0.7 mg/mL), (**E**) LDL 0.3 mg/mL alone, (F) HDL 0.3 mg/mL alone, and a combination of both α-syn 0.7 mg/mL + LDL 0.3 mg/mL (**G**) or HDL 0.3 mg/mL (**H**)

CSF HDL has been reported to have an intermediate size between serum HDL and serum low density lipoprotein (LDL) [26–28], which could modulate the inhibitory effect of CSF HDL. Thus, we also evaluated the inhibitory effect of serum LDL (LP2, Sigma-Aldrich) on α-syn aggregation to control for the difference in MW. We also evaluated TTR since this protein showed variations between the different CSF fractions. Under the in vitro α-syn aggregation conditions of the experiment summarised in Fig. 6, HDL concentration above 0.03 mg/mL completely blocked the spontaneous aggregation of α-syn, impeding the observation of subtleties of the inhibitory effect. Thus, we doubled the time of shaking (to 2 min) to further increase the spontaneous aggregation of α-syn in PBS. Under these slightly modified conditions, we observed aggregation of α-syn with 0.3 mg/mL HDL. However, there was no significant elongation phase as the fluorescence of the first (A1) and second (A2) plateau were low and very similar (Fig. 7A), confirming the high inhibitory power of HDL on α-syn spontaneous aggregation. Using the 2-min shaking assay conditions, we tested LDL and TTR at the same concentrations tested previously for HDL and evaluated their inhibitory effect based on the kinetic parameters extracted from the fluorescence traces (Fig. 7A-B). Surprisingly, LDL was a more potent inhibitor of spontaneous α-syn aggregation than HDL, since 0.3 mg/mL LDL completely blocked the aggregation in these conditions (low F_max_/A2 and > 200 h t2). TTR did not inhibit α-syn spontaneous aggregation when tested within the physiological concentration range (0.03 and 0.3 mg/mL) (Fig. 7A-B). Nevertheless, TTR showed partial inhibition, most noticeable in F_max_/A2 when tested at 1 mg/mL, which has been reported to be near the plasma physiological concentration of TTR [29]. These results suggest that the size, density, or lipidic content of the lipoprotein are not critical for the inhibition of α-syn aggregation, although these factors may modulate the level of inhibition on α-syn aggregation.

We have shown lipoprotein inhibition of α-syn aggregation by ThT, detection of oligomeric and amorphous species by conformational antibodies, and the measurement of monomeric α-syn by WB, but these techniques do not allow the observation of α-syn fibrillary species in the presence of lipoprotein. Thus, we used transmission electron-microscopy (TEM) to evaluate final products of aggregation reactions containing α-syn alone, α-syn with LDL or HDL, and HDL and LDL without α-syn (Fig. 7C-H). In agreement with ThT fluorescence traces and the kinetic parameters collected for these reactions, there was a marked decrease in the amount of α-syn fibrils in reactions with LDL and HDL compared to the α-syn alone reaction. Interestingly, the few small α-syn fibrils observed were intertwined with lipoproteins (Fig. 7G, H), which can be easily seen in the α-syn reaction with HDL (Fig. 7H). Similar structures, although with less clear intertwining, have been observed by co-incubating α-syn with NC-CSF (Supplementary Material Fig. S9).

### Solution NMR experiments on HDL, LDL and TTR

#### Impact of lipoprotein inhibition on diagnostic SAAs

**Fig. 8:**
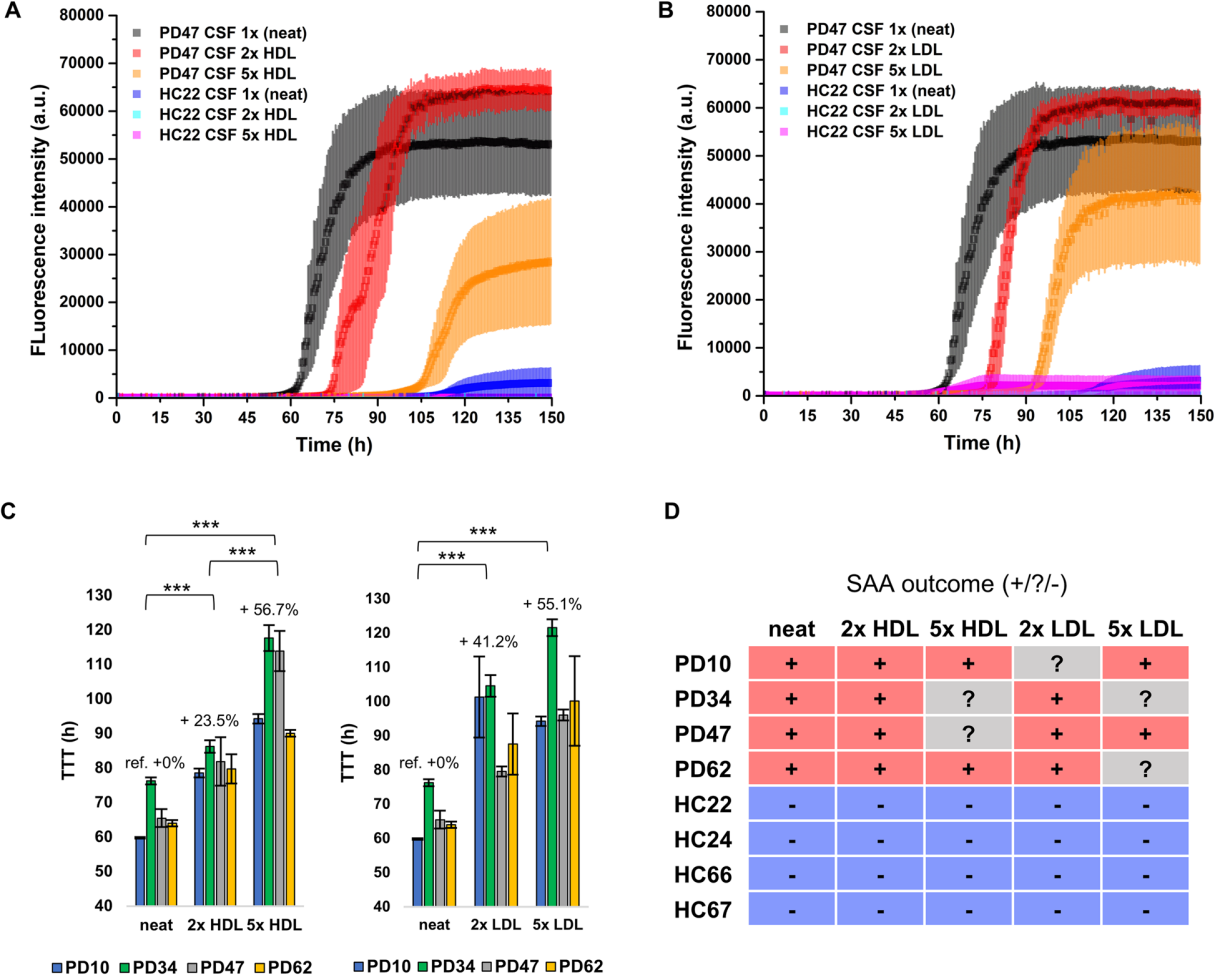
HDL and LDL significantly modulate α-syn aggregation in SAAs. **A-B** Representative SAAs traces performed on a neat PD (PD47) CSF sample and on a neat HC (HC22) CSF sample (1 × physiological concentration) and the same samples spiked with 0.006 mg/mL (2 × physiological concentration) and 0.024 mg/mL (5 × physiological concentration) HDL or LDL. The average kinetic traces with error bars representing the SEM calculated on three replicates of wells containing CSF additioned with HDL and LDL are shown in panels **A** and **B**, respectively. **C-D** Average time-to-threshold (TTT) values measured in all the PD samples. The average TTT and SEM were calculated by assuming a TTT of 125 h (maximum TTT observed) for replicates in which aggregation was not considered significant (F_max_ < 5000 a.u.). One-way ANOVA coupled with Tukey post-hoc test was applied to assess the statistical significance of the observed relative differences of all the individual measured traces among neat, 2 × HDL/LDL and 5 × HDL/LDL. Significant differences were marked with * with *** indicating a *p*-value <  < 0.001. **D** Summary of the final SAA outcome for the analysed PD and HC samples. The outcome was categorized as: positive ( +) when 3/3 replicates were determined positive by the probabilistic algorithm, inconclusive (?) when 2/3 replicates were determined positive by the probabilistic algorithm, and negative (-) when just 1/3 or 0/3 replicates were determined positive by the probabilistic algorithm

To determine if the inhibitory effect of lipoproteins on α-syn spontaneous aggregation also affects the seeded aggregation of α-syn intrinsic to SAAs, we used a highly sensitive and specific diagnostic α-syn SAA that can amplify α-syn seeds directly from PD/DLB/MSA CSF while showing no spontaneous aggregation with healthy control (HC) CSF [9, 19]. We supplemented *n* = 4 PD-CSF and *n* = 4 HC-CSF SAA reactions that have been tested consistently positive and negative in the SAA, respectively, with different levels of HDL and LDL. Each SAA reaction contains CSF, thus, we considered them to carry 1X the CSF physiological concentration of these proteins. Thus, 1X reactions are neat CSF reactions, while 2X and 5X reactions were supplemented with the purified lipoproteins to reach 2 and 5 times their respective CSF physiological concentration (Fig. 8). HDL partially inhibited the amplification of PD α-syn seeds, and it slowed down the amplification reaction. The time to reach the fluorescence threshold of 5000 a.u. (TTT) was ~ 24% longer on average in the reaction supplemented to 2X HDL (Fig. 8C). This inhibition was dose dependent, as 5X HDL had a greater impact on the amplification of PD α-syn seeds; since the TTT increased by ~ 55% on average with respect to those measured in neat samples. More importantly, 5X HDL was able to convert two consistently positive ( +) PD-CSF samples (PD34 and PD47) into an inconclusive (?) result [19] (Fig. 8D). LDL followed a similar pattern as HDL, causing a progressively longer TTT at increasing concentration of LDL. At 2X LDL the SAA outcome changed from “ + ” to “?” for one PD sample (PD10), while at 5X for two PD samples changed (PD34 and PD62). We also evaluated if HDL could cause some cross-reaction and induce spontaneous aggregation in HC samples, however there was no relevant increase in fluorescence in reactions containing HDL or LDL as compared to the control condition. The effect of adding 2X and 5X HSA and TTR was also tested in one HC sample (HC22) and in one PD sample (PD47, experiments summarised in Supplementary Material Table S4). In agreement with previous results, HSA and TTR did not significantly inhibit the amplification of PD α-syn seeds and did not induce any spontaneous aggregation in the HC sample.

### α-Syn aggregation experiments performed on ApoA1- and ApoE-immunodepleted pooled CSF

**Fig. 9:**
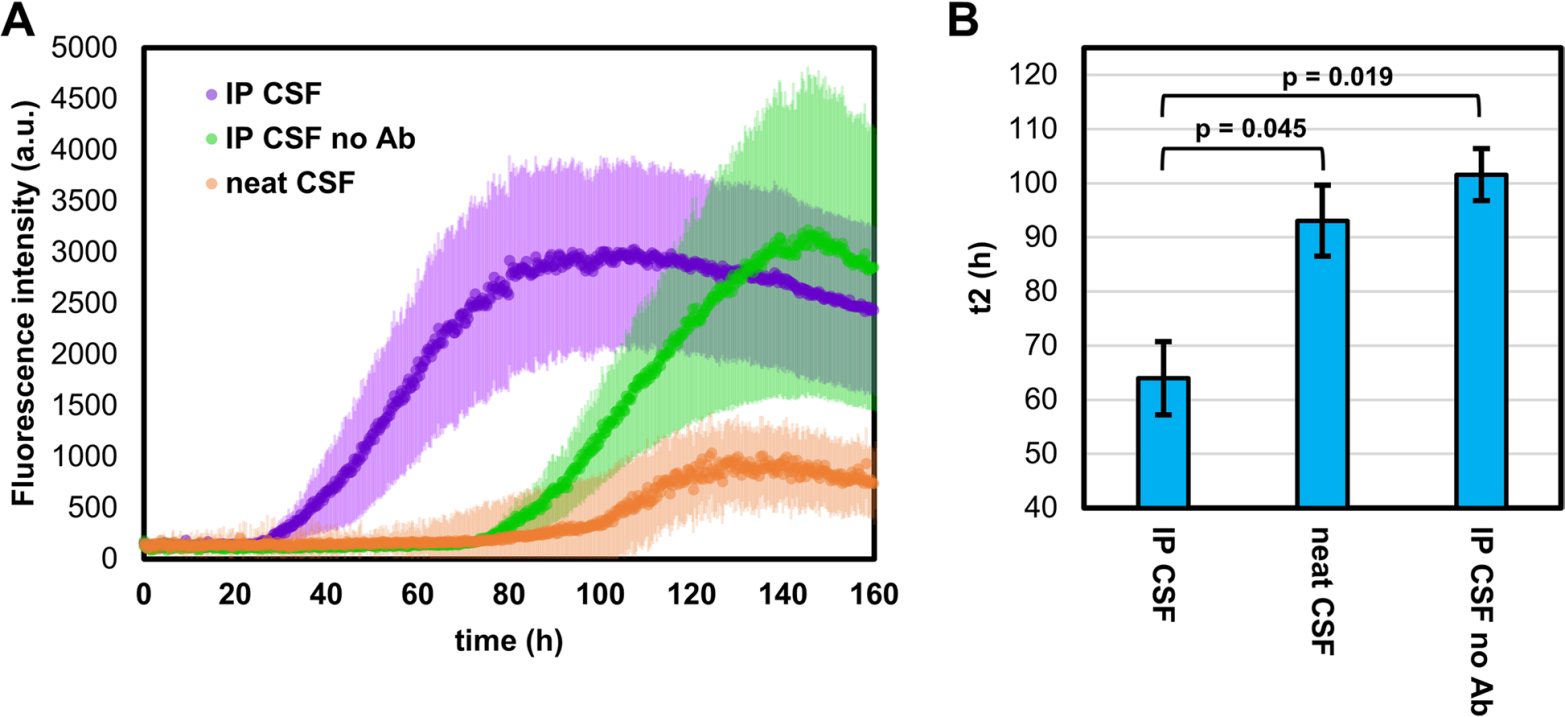
Experiments on ApoA1- and ApoE-immunodepleted CSF. **A** Protein aggregation assay performed using 0.7 mg/mL of recombinant α-syn in PBS with 40 μL of: CSF subjected to ApoA1 and ApoE IP (IP CSF), CSF subjected to IP without antibodies (IP CSF no Ab) and neat CSF belonging to different aliquots of the same NC pool. To remove the background fluorescence, the average fluorescence of three replicates containing the same reaction mix without α-syn was subtracted prior to the analysis. All ThT fluorescence traces are represented as average intensity over 3 replicates with error bars representing the SEM. **B** Average t2 fitted parameters with error bars representing the SEM. *P*-values were calculated by applying one-way ANOVA followed by Tukey post-hoc test

To further confirm the role of lipoproteins in the patient-dependent CSF inhibition on α-syn aggregation, we depleted ApoA1 and ApoE from a newly made pooled CSF (see Materials and Methods Sect. 2.3, 2.11, and Supplementary Fig. S11) by immunoprecipitation (IP). We then analysed α-syn spontaneous aggregation in the presence of immunodepleted CSF (IP CSF), CSF subjected to the IP procedure without antibodies (IP CSF no Ab), and neat CSF (neat CSF) from the same pooled CSF sample. The results of this experiment are summarised in Fig. 9. As expected, depletion of the two most abundant CSF apolipoproteins (IP CSF) resulted in a significantly faster α-syn spontaneous aggregation as compared with IP CSF no Ab and neat CSF.

### CSF ApoA1, ApoE and total protein levels correlations with SAA kinetic parameters

**Fig. 10:**
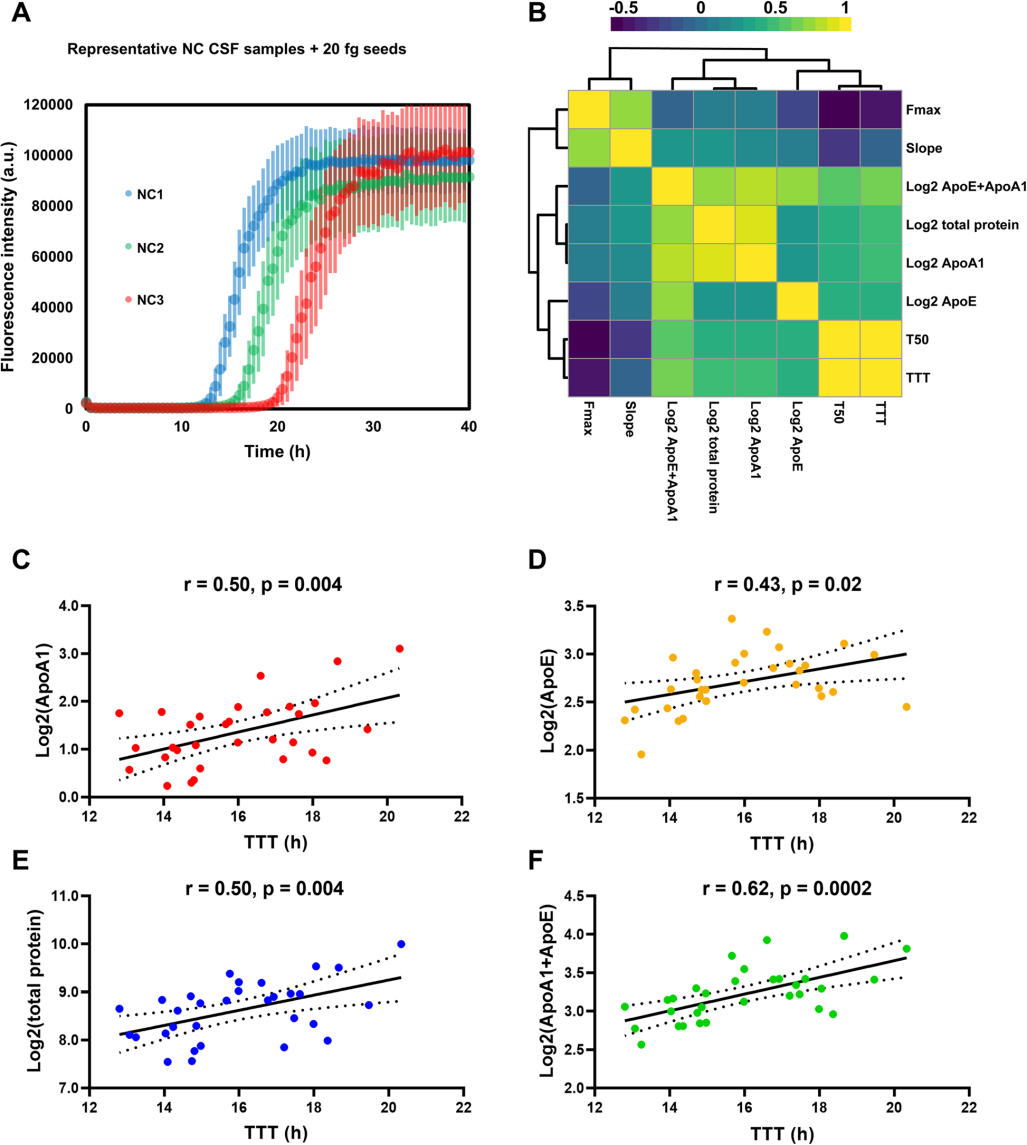
CSF ApoA1, ApoE and total protein content significantly correlate with SAA time variables. **A** representative SAA ThT fluorescence traces relative to samples producing the shortest (NC1, TTT = 12.8 h), median (NC2, TTT = 15.8 h) and longest (NC3, TTT = 20.3 h) TTT averaged on three replicates. All ThT fluorescence traces are represented as average intensity over 3 replicates with error bars representing the SEM. **B** Heatmap summarizing correlations (Pearson’s) between SAA kinetic parameters and Log2-transformed CSF ApoA1, ApoE, ApoA1 + ApoE, and total protein. Hierarchical clustering was performed by using Ward linkage criterion. **C-F** scatter plots with detail of Pearson’s correlation coefficients and relative p-value for TTT vs Log2-transformed CSF concentrations (originally in μg/mL) of ApoA1 **(C)**, ApoE **(D)**, total protein **(E)**, and ApoA1 + ApoE **(F)**. Linear regression lines with their 95% confidence intervals (dotted lines) are also displayed

Lastly, we measured levels of the two most abundant CSF apolipoproteins (ApoA1 and ApoE) in *n* = 31 SAA-negative NC CSF samples. Although albumin represents most of total CSF proteins, total protein measurements were also considered, as both CSF ApoA1 and albumin derive from peripheral blood [30] and we expected their levels to be highly correlated. The NC CSF samples were spiked with α-syn seeds (20 fg) and retested with SAA to analyse possible correlations between the levels of these analytes and SAA kinetic parameters. The results of these experiments are summarised in Fig. 10. After the addition of 20 fg α-syn seeds, all the NC CSF samples tested SAA-positive. Considering the fact that SAA time variables (TTT and T50) should be theoretically linearly related to the logarithm of α-syn seed mass [12], we preferred to use Log2-transfomed total protein, ApoA1 and ApoE levels to calculate correlation coefficients and to perform linear regressions. However, similar results were found when logarithm transformation was not applied. As can be seen from the correlation heatmap present in Fig. 10B, CSF ApoA1 and CSF total protein were highly correlated. Moreover, ApoA1, ApoE and total protein were found significantly correlated to SAA T50 and TTT (r = 0.4–0.5, FDR-adjusted *p*-value < 0.05). Interestingly, Log2-transfomed sum of ApoA1 and ApoE was the variable showing the highest correlation with T50 (r = 0.55) and TTT (r = 0.62), which is consistent with the fact that lipoproteins in general can modulate α-syn aggregation in SAAs.

## Discussion

Inhibition of spontaneous and seeded fibrilization of α-syn by human CSF has been briefly reported in recent literature, but not yet characterized [1, 11–13]. Given the potential interference of CSF-related inhibition in diagnostic SAAs, we studied this phenomenon in a multi-technique approach, including nLC-nESI HRMS/MS, dot-blot, ELISA, IP, WB, TEM, solution NMR, a highly accurate and standardized diagnostic SAA, and different in vitro aggregation conditions to evaluate spontaneous aggregation of α-syn. We first learned that the inhibitory effect of CSF is donor dependent, which added a new layer of complexity to this issue. Using α-syn aggregation conditions that exacerbated the inhibitory effect of CSF on both spontaneous and seeded aggregation at physiological pH and ionic strength, we found a dramatic difference in the inhibitory effect of CSF from two NPH cases. NMR and MS studies showed that the most inhibitory NPH sample presented higher protein and lipoprotein levels. After fractionating a pool of human NC CSF using MWCO filters, we confirmed that HMW components (> 100 kDa) retained the same inhibitory effect on α-syn aggregation as whole CSF, while fractions of smaller MW were less inhibitory. It should be considered that the pore size distribution of MWCO filters allows a low percentage of HMW components to pass through the filter in smaller MW fractions. This potential leak of HMW material into lower MW fractions could explain the lower but still detectable inhibitory effect observed in those fractions. Using MS we identified albumin, TTR, PGDS, and apolipoproteins to be the most abundant HMW components in the NC CSF pool used. The distribution of apolipoprotein in the CSF fractions correlated with the inhibition of each fraction on α-syn aggregation, while albumin, TTR, and PGDS did not. Apolipoproteins, particularly ApoA1 (28.3 kDa) and ApoE (34 kDa), were the most represented in the > 100 kDa fraction. Given the MW, these apolipoproteins were most likely assembled in lipoproteins in CSF. We then confirmed that serum-purified HDL in the absence of CSF milieu was able to inhibit α-syn spontaneous aggregation. Moreover, the partial inhibition was observed when evaluating HDL at CSF sub-physiological concentrations. These findings were confirmed by tracking the aggregation using dot blot with two different conformational antibodies and WB detecting the monomeric α-syn that was not converted into fibrils in the aggregation reaction. In these experiments, although most α-syn remained monomeric at 1 mg/mL HDL, we could spot the presence of a band at 150–200 kDa, suggesting that high HDL concentrations might have stabilised some prefibrillar oligomers, impeding their conversion into fibrils. Since CSF HDL are bigger in size than blood HDL and smaller than blood LDL [27, 28], we also evaluated the inhibitory effect of serum-purified LDL on α-syn aggregation and observed a similar or greater inhibitory effect than with HDL even at sub-physiological concentrations. We evaluated HDL and LDL at the same concentrations in terms of mg/mL, but because of the smaller molecular weight of HDL, molar concentration of HDL was greater than the concentration of LDL in our α-syn aggregation experiments. Thus, the higher inhibitory effect of LDL might indicate that the inhibition is driven by the lipidic fraction of the complex, although there are many reports showing that lipids promote aggregation of α-syn [31].

We used TEM to observe the ultrastructure of the α-syn fibrils in the presence of HDL and LDL and found both lipoproteins to colocalize with the fibrils, suggesting a direct interaction with α-syn aggregates. Although solution NMR has previously proven to be a suitable tool to identify proteins interacting with monomeric α-syn [16, 32], we could not find any evidence of interactions between HDL or LDL with monomeric recombinant α-syn. Hypothesizing an interaction between HDL and oligomeric or prefibrillar species would be compatible with those results. The α-syn aggregation conditions in PBS showed a peculiar double sigmoidal behaviour. TEM analysis found the first plateau to be enriched in prefibrillar/oligomeric species and the second plateau enriched in fibrils, which has also been reported for other amyloidogenic proteins [33]. HDL primarily reduced the formation of oligomeric/protofibrillary species in the first plateau, which slowed down or completely blocked the subsequent formation of α-syn fibrils in a dose dependent manner starting at sub-physiological concentrations. Conversely, depleting the two most abundant CSF apolipoproteins (ApoA1 and ApoE) from CSF resulted in faster spontaneous aggregation of α-syn in PBS. Overall, these results are consistent with an inhibitory effect on the formation of oligomeric species exerted by CSF lipoproteins, especially in the lag-phase before the first plateau.

We then evaluated the effect of lipoproteins on the amplification of α-syn seeds from human PD CSF samples in an ultrasensitive SAA. In these experiments, the reactions were supplemented with HDL and LDL to mimic CSF samples with 2X or 5X the physiological levels of HDL and LDL. We found that both HDL and LDL could significantly change the kinetic parameters of the amplification reaction starting from 2X concentration. At 2X LDL concentration, the SAA outcome of one of four PD samples changed from positive to inconclusive, while the same happened for two of four PD samples at both 5X HDL and LDL. Nevertheless, the likelihood of encountering CSF samples containing such high levels of lipoproteins is low, which is in line with the impressive sensitivity and specificity consistently reported by diagnostic SAAs [4]. However, the concentration of all lipoproteins combined, in addition to other lesser inhibitory CSF components, could explain the 5% of the clinical and pathologically confirmed PD/DLB cases that are usually negative when tested by SAAs. We also demonstrated that, in a small cohort of NC CSF samples spiked with 20 fg of α-syn seeds, SAA T50 and TTT were significantly correlated to CSF ApoA1 and ApoE levels and even more correlated to their sum. Additionally, CSF total protein, a measurement which is readily available for most patients who undergo CSF analysis for a diagnostic workup, was found to be strongly correlated to CSF ApoA1 levels and moderately correlated with T50 and TTT kinetic parameters. Our results confirm that the kinetics of α-syn seed amplification is not only a function of the mass of α-syn seeds or the α-syn “strain”, but also of the overall composition of the CSF, and particularly the concentration of lipoproteins/apolipoproteins. Our findings may have profound implications for the use of kinetic parameters in SAA assays to predict disease status, disease progression, and disease prognosis, since we demonstrated that the inhibitory effect of CSF is donor dependent. It remains unknown if disease progression correlates with the amount of seeds in a CSF sample, but if it does, estimation of α-syn seed levels by kinetic parameters will need models to control for CSF components such as apolipoproteins or total protein as a surrogate measure. Indeed, in the absence of matrix effects, TTT and T50 should be linearly associated with the logarithm of the aggregated α-syn mass [12]. The addition of CSF lipoprotein/total protein content as a covariate can restore this linear relationship, thus allowing the quantification of aggregated α-syn mass. In addition to our results, α-syn species and lipoproteins have already been shown to interact by co-immunoprecipitation both in plasma [34] and CSF [35]. Given that apolipoprotein levels and genotype have been previously documented as relevant in PD [36, 37] and that, in our experiments, lipoproteins inhibited α-syn aggregation in vitro at subphysiological concentrations, the interaction between α-syn and lipoproteins might be biologically relevant and deserves further investigation.

## Conclusions

Our results describe a novel interaction between lipoproteins and α-syn that inhibits the formation of α-syn fibrils and could have relevant biological implications in vivo. Our findings also have direct and important implications for the future development and improvement of α-syn SAAs, which are currently the most promising diagnostic tool for synucleinopathies. The donor-specific inhibition of CSF on α-syn aggregation reported here offers an explanation for the lack of correlation seen between kinetic parameters and clinical progression. Moreover, our data reveals apolipoprotein as the main inhibitory components of CSF, suggesting that measurements of apolipoproteins and/or total protein content could be incorporated into data analysis models to eliminate confounding effects of CSF milieu on α-syn seed quantification efforts using kinetic parameters.

## Supplementary Information

**Additional file 1:** **Fig. S1.** Silver staining on α-syn used in Protein aggregation experiments. Two replicate silver staining experiments performed on a 4-20% SDS-PAGE gel with 1 μg of purified α-syn after one (lane 3) and two (lane 2) size-exclusion chromatography steps. **Fig. S2.** T_50_ measured in Six different human CSF samples spiked with 20 fg of seeds. The measured T_50_ parameters were globally different, as assessed by one-way analysis of variance (ANOVA) and Fisher’s LSD post-hoc test for mean comparisons. *0.01<*p*<0.05; **0.001<*p*<0.01: ***p<0.001. **Fig. S3.** Quantitative regression analysis of the seed masses in the absence of human CSF. Measured t2 parameters for the seeded experiment vs the quantity of seeds added; the horizontal axis is displayed in log10 scale. The t2 values displayed result from the average of three replicates, error bars reflect the standard deviation of the mean value. The data were fitted with a natural log function, the correlation between t2 and the added seed masses was assessed by means of Pearson’s correlation coefficient (r). **Fig. S4.** NMR titrations of α-syn with CSF fractions. Intensity decreases of the signals of two-dimensional (2D) ^15^N–^1^H HSQC experiments acquired at 950 MHz at T = 283 K on ^15^N labelled α-syn (100 μM) in PBS after the addition of: (A) whole pooled CSF in PBS, (B) < 3 kDa CSF fraction in PBS and (C) > 100 kDa CSF fraction in PBS. The residues experiencing the largest decreases in signal intensity (smaller by one or more standard deviations with respect to the average value) are highlighted in light blue. The intensity ratios corresponding to overlapping peaks are highlighted in red (their values were not considered in the calculation of the average decreases and standard deviations). **Fig. S5.** CSF pH drift. The pH change due to the exposure of CSF to air was monitored over time in 500 μL of undiluted pooled CSF (A) and in the presence of PBS (400 μL CSF + 200 μL PBS 3x) in polypropylene vials with a Thermo Scientific Orion pH-meter equipped with a glass 6 mm diameter pHenomenal MIC 220 Micro electrode. Right before each measurement, the sample was vortexed for 20 sec and left open to air for another 20 sec. **Fig. S6.** Different CSF fractions differently affect α-syn aggregation. Mean fitted t2 parameters of samples with 40 μl of PBS/CSF fractions. The values displayed result from the average of three replicates with error bars reflecting the SEM. For whole CSF and the >100 kDa fraction the total duration of the experiment is shown due to the absence of appreciable aggregation. **Fig. S7.** Raw images of the dot-blot assays. A-B) Native images of the dot-blot assay performed on α-syn alone replicates at different timepoints with OC (A) and A11 (B) conformational antibodies. C-D) Native image of the dot-blot assay performed on the HSA and HDL containing samples with OC (C) and A11 (D) conformational antibodies. **Fig. S8.** WB experiments to track α-syn aggregation in the presence of human HDL. A) α-Syn aggregation patterns in samples collected at different timepoints of the spontaneous aggregation process, was monitored by WB using Syn211 antibody (4-20% SDS-PAGE, 2 μg protein loaded). Monomeric α-syn decreases as t increases due to the formation of fibrils. B) In a similar way, a WB with Syn211 was performed on the reaction products obtained after 180 h, at different HDL concentrations with and without α-syn (exposure time 210 s). C) The experiment was then repeated by doubling the amount of sample loaded into the gel to better highlight the presence of oligomeric species (exposure time 30 s). Under these conditions, chosen to better visualize the signal at 150-200 kDa, the α-syn monomer bands at 1 and 0.3 mg/mL HDL may not quantitively reflect monomer concentration (overloaded lanes). **Fig. S9.** Representative TEM images of α-syn incubated with CSF. Representative TEM images obtained by analyzing samples obtained by the co-incubation of α-syn 0.7 mg/mL at 37 °C with pooled human CSF (1:5 ratio with respect to total reaction volume). Samples were subjected to cycles of incubation (13 min) and shaking (double-orbital, 2 min) at 500 rpm. **Fig. S10.** NMR titrations of α-syn with HDL, LDL and TTR. A) Intensity decreases of the signals of two-dimensional (2D) ^15^N–^1^H HSQC experiments acquired at 950 MHz at T = 283 K on ^15^N labelled α-syn (100 μM) in PBS after the addition of 0.57 mg/mL serum-derived HDL. The intensity ratios corresponding to overlapping peaks are highlighted in red. B) Intensity decreases of the signals of two-dimensional (2D) ^15^N–^1^H HSQC experiments acquired at 950 MHz at T = 283 K on ^15^N labelled α-syn (100 μM) in PBS after the addition of 1 mg/mL serum-derived LDL. C) Intensity decreases of the signals of two-dimensional (2D) ^15^N–^1^H HSQC experiments acquired at 950 MHz at T = 283 K on ^15^N labelled α-syn (100 μM) in PBS after the addition of 3 mg/mL TTR. **Fig. S11.** WB experiments performed on immunodepleted CSF. WB experiments were performed with anti-ApoA1 (MIA1404) and anti-ApoE (PA5-27088) antibodies on neat CSF and supernatants (400 μL CSF, 100 μL slurry) resulting from immunoprecipitation procedure performed using different quantities of the same antibodies (IP CSF samples), immunoprecipitation performed without antibodies (CSF IP no Ab). The conditions relative to IP CSF ApoA1 60 µg and IP CSF ApoE 20 µg were then selected for Protein aggregation assays. **Table S1.** Concentration factors and final volumes of the CSF fractions.

## Data Availability

All the relevant data generated or analysed during this study are included in this published article and its supplementary information files. Raw fluorescence and mass spectrometry data are available to the corresponding authors upon reasonable request.

## Abbreviations

CV: Coefficient of variation
DLB: Dementia with Lewy bodies
EDTA: Ethylenediaminetetraacetic acid
ELISA: Enzyme-linked immunosorbent assay
emPAI: Exponentially modified PAI
FDR: False discovery rate
Fmax: Maximum fluorescence
Fmin: Minimum fluorescence
HC: Healthy control
HDL: High-density lipoprotein
HMW: High molecular weight
HSA: Human serum albumin
HSQC: Heteronuclear single quantum correlation
IMAC: Immobilized metal ion affinity chromatography
IP: Immunoprecipitation
IPTG: Isopropil-β-D-1-tiogalattopiranoside
LB: Luria–Bertani
LDL: Low-density lipoprotein
MSA: Multiple system atrophy
MW: Molecular weight
MWCO: Molecular weight cut-off
NC: Neurological controls
nLC-nESI HRMS/MS: NanoLiquidChromatography coupled to High Resolution Mass Spectrometry equipped with a nanoelectrospray interface
NMR: Nuclear magnetic resonance
NPH: Normal pressure hydrocephalus
OD600: Optical density at 600 nm wavelength
PBS: Phosphate buffered saline
PD: Parkinson's disease
PGDS: Prostaglandin-D synthase
PIPES: Piperazine-N,N′-bis(2-ethanesulfonic acid)
PMCA: Protein misfolding cyclic amplification
PMSF: Phenylmethylsulfonyl fluoride
PVDF: Polyvinylidene fluoride
RFU: Relative fluorescence units
ROI: Region of interest
RT-QuIC: Real-time quaking-induced conversion
SAA: Seed amplification assay
SDS-PAGE: Sodium Dodecyl Sulphate—PolyAcrylamide Gel Electrophoresis
SEM: Standard error of the mean
T50: Time to reach the half maximum
TEM: Transmission electron microscopy
ThT: Thioflavin-T
TTR: Transthyretin
WB: Western blot
α-syn: α-Synuclein
