# Supplementary material for: Cerebrospinal fluid lipoproteins inhibit α-synuclein aggregation by interacting with oligomeric species in seed amplification assays
Source: Mol Neurodegener. 2023 Apr 1;18:20. doi: 10.1186/s13024-023-00613-8 (PMC10068178; doi:10.1186/s13024-023-00613-8)
Supplement: Supplementary file 1 — Additional file 1: Fig. S1. Silver staining on α-syn used in Protein aggregation experiments. Two replicate silver staining experiments performed on a 4-20% SDS-PAGE gel with 1 μg of purified α-syn after one (lane 3) and two (lane 2) size-exclusion chromatography steps. Fig. S2. T50 measured in Six different human CSF samples spiked with 20 fg of seeds. The measured T50 parameters were globally different, as assessed by one-way analysis of variance (ANOVA) and Fisher’s LSD post-hoc test for mean comparisons. *0.01<p<0.05; **0.001<p<0.01: ***p<0.001. Fig. S3. Quantitative regression analysis of the seed masses in the absence of human CSF. Measured t2 parameters for the seeded experiment vs the quantity of seeds added; the horizontal axis is displayed in log10 scale. The t2 values displayed result from the average of three replicates, error bars reflect the standard deviation of the mean value. The data were fitted with a natural log function, the correlation between t2 and the added seed masses was assessed by means of Pearson’s correlation coefficient (r). Fig. S4. NMR titrations of α-syn with CSF fractions. Intensity decreases of the signals of two-dimensional (2D) 15N–1H HSQC experiments acquired at 950 MHz at T = 283 K on 15N labelled α-syn (100 μM) in PBS after the addition of: (A) whole pooled CSF in PBS, (B) < 3 kDa CSF fraction in PBS and (C) > 100 kDa CSF fraction in PBS. The residues experiencing the largest decreases in signal intensity (smaller by one or more standard deviations with respect to the average value) are highlighted in light blue. The intensity ratios corresponding to overlapping peaks are highlighted in red (their values were not considered in the calculation of the average decreases and standard deviations). Fig. S5. CSF pH drift. The pH change due to the exposure of CSF to air was monitored over time in 500 μL of undiluted pooled CSF (A) and in the presence of PBS (400 μL CSF + 200 μL PBS 3x) in polypropylene vials with a Thermo Scientific [file 13024_2023_613_MOESM1_ESM.docx]

**Supplementary Material for**

**Cerebrospinal fluid lipoproteins inhibit α-synuclein aggregation by interacting with oligomeric species in seed amplification assays**

Giovanni Bellomo^1*^, Silvia Paciotti^1^, Luis Concha-Marambio^2^, Domenico Rizzo^3,4^, Anna Lidia Wojdaƚa^1^, Davide Chiasserini^5^, Leonardo Gatticchi^5^, Linda Cerofolini^3,6^, Stefano Giuntini^3^, Chiara Maria Giulia De Luca^7^, Yihua Ma^2^, Carly M. Farris^2^, Giuseppe Pieraccini^8^, Sara Bologna^3^, Marta Filidei^1^, Enrico Ravera^3,4,6^, Moreno Lelli^3,4,6^, Fabio Moda^7^, Marco Fragai^3,4,6^, Lucilla Parnetti^1^ and Claudio Luchinat^3,4,6*^.

^1^Laboratory of Clinical Neurochemistry, Section of Neurology, Department of Medicine and Surgery, University of Perugia; Piazzale Lucio Severi 1/8, Perugia, 06132, Italy.

^2^R&D Unit, Amprion Inc., 11095 Flintkote Av., San Diego, San Diego, 92121, CA, USA

^3^Magnetic Resonance Center (CERM), University of Florence, Via Luigi Sacconi 6, Sesto Fiorentino, 50019, Italy.

^4^Department of Chemistry “Ugo Schiff”, University of Florence, Via della Lastruccia 3, Sesto Fiorentino, 50019, Italy.

^5^Section of Physiology and Biochemistry, Department of Medicine and Surgery, University of Perugia, Perugia, Piazzale Lucio Severi 1/8, Perugia, 06132, Italy.

^6^Consorzio Interuniversitario Risonanze Magnetiche Metallo Proteine (CIRMMP), Via Luigi Sacconi 6, Sesto Fiorentino, 50019, Italy.

^7^Fondazione IRCCS Istituto Neurologico Carlo Besta, Division of Neurology 5 and Neuropathology, Via Celoria 11, Milano, 20133, Italy.

^8^CISM Mass Spectrometry Centre, Department of Health Sciences, University of Florence, Viale Gaetano Pieraccini 6, Firenze, 50139 Italy.

*To whom correspondence may be addressed

**Email:**  [giovanni.bellomo@unipg.it](mailto:giovanni.bellomo@unipg.it), [luchinat@cerm.unifi.it](mailto:luchinat@cerm.unifi.it)

**This PDF file includes:**

Supplementary Materials and Methods

Supplementary Results

Figures S1 to S11

Tables S1 to S4

Supplementary References

**Supplementary Materials and Methods**

**Solution NMR experiments**

All the NMR spectra were acquired at 283 K with a Bruker Avance III HD NMR spectrometer operating at 950 MHz ^1^H Larmor frequency, equipped with a cryogenically cooled probe. For all the measurements performed, samples were analysed in 3 mm diameter NMR tubes. Spectra were initially processed with the Bruker TOPSPIN 4.0 software tools.

Solution NMR experiments on NPH CSF

1D NMR experiments (Bruker sequence: zgesgp, number of scans = 128) using NPH CSF were performed by analysing 170 μL of CSF samples + 10 μL D_2_O (5.6%).

Solution NMR titrations with monomeric α-syn

2D ^1^H-^15^N HSQC spectra (Bruker sequence: hsqcetfpf3gp, number of scans = 16) were performed to test possible interactions between monomeric α-syn and CSF fractions/HDL/LDL/TTR.

Solution NMR titration experiments were carried out starting from 200 μL of a solution containing 100 μM of recombinant ^15^N labelled α-syn in PBS buffer and 10% D_2_O.

For CSF fractions titration experiments, the added volumes were 100 μL for fraction 1, 5 and 6, while 62.5 μL, 71 μL and 83 μL were added for fraction 2, 3 and 4 respectively to compensate for the different concentration factor with respect to fraction 5.

For HDL titration experiments 2 and 10 μL of a solution containing 11.97 mg/mL of human serum HDL (Sigma Aldrich, LP3) were added to the solution containing monomeric α-syn.

For LDL titration experiments 2, 10 and 40 μL of a solution containing 6.27 mg/mL of human serum LDL (Sigma Aldrich, LP2) were added to the solution containing monomeric α-syn.

For TTR titration experiments 20 and 40 μL of a solution containing 30 mg/mL of recombinant human TTR were added to the solution containing monomeric α-syn.

In order to compute amplitude ratios and/or evaluate chemical shifts perturbations, 2D ^1^H-^15^N heteronuclear single quantum correlation (HSQC) spectra were further analysed by the program Computer Aided Resonance Assignment [23].

**Mass spectrometry measurements**

The analyses of proteins contained in CSF and CSF fractions were performed by nano Liquid Chromatography coupled to High Resolution Mass Spectrometry equipped with a nanoelectrospray interface (nLC-nESI HRMS/MS). From each CSF fraction a 10 µL volume was taken in triplicate and reduced with DTT (1 µL of a 0.5 mg/mL solution in ultrapure water) for 30 min at room temperature in the dark and then alkylated adding 1 µL of a 2.5 mg/mL iodoacetamide solution in ultrapure water and left for 20 min at RT in the dark. The samples were diluted with four volumes of 50 mM NH_4_HCO_3_ pH 8. Protein digestion was started by adding 1 µL of 0.4 mg/mL LysC solution and incubating for 3 h at 37 °C, then adding 1 µL of 0.5 mg/mL sequencing grade trypsin (Promega) solution and incubating at 37 °C overnight. The reaction was stopped by adding 1 µL 10% trifluoroacetic acid to reach a < 2.5 pH value. C18 Empore (3M) Stage tips were prepared in-house and sequentially conditioned with 50 µL methanol, 50 µL 80% acetonitrile, 19.5% ultrapure water, 0.5% acetic acid and then with 50 µL 0.5% acetic acid. The digested samples were loaded into the stage tips, washed with 50 µL 0.5% acetic acid, then dried and peptides eluted with 50 µL methanol, 50 µL 80% acetonitrile, 19.5% ultrapure water, 0.5% acetic acid. The samples were then concentrated to less than 10 µL and brought to a final volume of 20 µL with 0.5% acetic acid; a 1 µL volume was injected into the nLC-nESI HRMS/MS system; this was composed of a nanoLC system EASY-nLC 1200 coupled to a LTQ Orbitrap hybrid mass spectrometer (Thermo Scientific). The nESI spray potential was 1.7 kV, capillary and tube lens voltages were 42 and 120 V, respectively. The chromatographic column was an Acclaim^®^ PepMap 100 C18, 3 µm, 100 Å, 75 µm x 150 mm, operating at 300 nL/min flow rate. Solvent A was 100 % water and solvent B was 80% acetonitrile/20% water, both containing 0.1% formic acid; solvents were of LC-MS grade from Sigma (Sigma Italy, Merck). Elution was done by gradient starting from 2% B for 5 min, to 40% B in 340 min, to 90% B in 5 min and then returned to initial conditions. Data were acquired in data dependent manner, performing a survey HRMS full scan from 350 to 2000 m/z at 60000 nominal resolution (at m/z 400) in the Orbitrap, using a 1 x 10^6^ target value. Precursors were isolated from the seven most intense signals above 500 a.u. threshold with an isolation window of 2 Da. Normalized collision energy of 35% and an activation time of 20 ms were used. Precursor ions with no charge state assigned and singly charged did not trigger MS/MS experiments. Precursor masses already selected were dynamically excluded for 30 s with an exclusion window of 20 ppm (repeat count 2, repeat duration 15 s). The acquired data were searched with Mascot 2.4 search engine (Matrix Science Ltd., London, UK) against a human database created from NCBI. Searches were performed allowing: (i) trypsin as enzyme, (ii) up to two missed cleavage sites, (iii) 10 ppm of tolerance for the monoisotopic precursor ion and 0.5 mass unit for monoisotopic fragment ions, (iv) carbamidomethylation of cysteine and oxidation of methionine as variable modifications. A target-decoy search was used: a false discovery rate (FDR) of 1% was imposed and the criterion used to accept protein identification included probabilistic score sorted by the software. The exponentially modified PAI (emPAI) values were used to estimate protein abundances [24,25]. Considering that the emPAI score can be considered approximately proportional to the mass/volume concentration of the compound, in order to make comparisons among fractions, we multiplied the emPAI score with the molecular weight (MW) in kDa to obtain a value proportional to the mass/volume concentration.

**Transmission electron microscopy**

The final reaction products of SAA for samples containing α-syn 0.7 mg/mL, LDL 0.3 mg/mL, α-syn 0.7 mg/mL + LDL 0.3 mg/mL, α-syn 0.7 mg/mL + HDL 0.3 mg/mL and α-syn 0.7 mg/mL + pooled human CSF (1:5 ratio) were diluted 3-fold in water. Ten µL of each dilution was adsorbed onto 200-mesh Formvar-carbon coated nickel grids, at room temperature. After 30 minutes, the remaining drop was dried using filter paper and the grids were stained with 25% Uranyl Acetate Replacement (negative staining) for 10 min and air-dried for 15 minutes. Images were taken using a FEI Tecnai Spirit electron microscope (120 kV), equipped with an Olimpus Megaview G2camera.

**Supplementary Results**

**Solution NMR experiments on CSF fractions**

To clarify the molecular basis of the inhibition of α-syn aggregation by CSF, we titrated ^15^N isotopically enriched monomeric α-syn (1.44 mg/mL, 100 μM) with all the CSF fractions and evaluated the intensity changes in α-syn amide resonances by 2D ^1^H-^15^N HSQC solution NMR spectra. Relevant intensity decreases were observed by titrating α-syn with whole CSF and with the <3 kDa fraction (Fig. S4A and S4B), while no relevant intensity decreases were detected for the other fractions (as an example, the results for the >100 kDa fraction are shown in Fig. S4C). However, the intensity decreases observed by titrating α-syn with whole CSF and with the <3 kDa fraction were accompanied by a shift in the position of the amide-proton of histidine-50 (for whole CSF Δ^1^H = 0.042 ppm, Δ^15^N = 0.26 ppm). The resonance frequency of histidine-50 amide protons of α-syn is known to be particularly sensitive to pH changes, which also affect the chemical exchange between bulk water protons and amide protons, which in turn modulates the intensity of peaks in 2D ^1^H-^15^N HSQC solution NMR spectra. To test whether CSF could produce such a pH change also in the presence of PBS, we performed pH measurements over-time on the whole CSF fraction (2/3 CSF + 1/3 PBS 3x) and in undiluted CSF. The results of these experiments are shown in Fig. S5: exposing CSF to air upon vigorous shaking caused a significant increase in pH both in the presence and absence of PBS. This effect had been reported previously (1, 2) and is produced by the loss of carbon dioxide from CSF (and the consequent displacement of the CO_2_/HCO_3_^–^ equilibrium). This effect is likely responsible for the intensity decreases observed in HSQC spectra while titrating α-syn with whole CSF and with the <3 kDa fraction. It is also well known that the aggregation propensity of α-syn is strongly dependent on pH (3). For this reason, we interpreted the slight decrease in the maximum fluorescence signal observed for the <3 kDa fraction (main text Fig. 3B), as a possible effect of the pH increase due to the loss of CO_2_, which could be significantly present both in this fraction and in the whole CSF sample (the other fractions were eluted multiple times with PBS).

**Supplementary Figures**


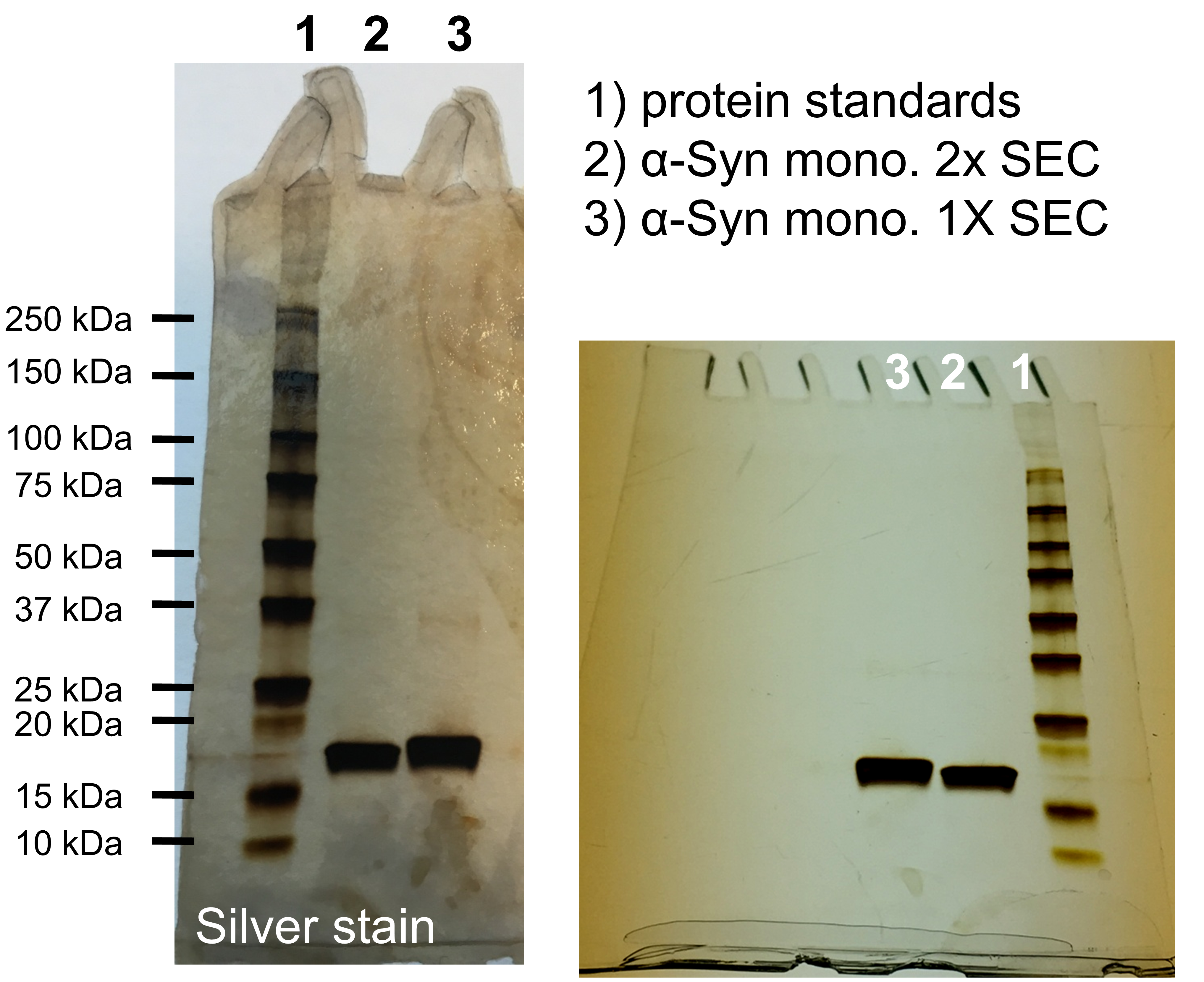


## Fig. S1. Silver staining on α-syn used in Protein aggregation experiments. Two replicate silver staining experiments performed on a 4-20% SDS-PAGE gel with 1 μg of purified α-syn after one (lane 3) and two (lane 2) size-exclusion chromatography steps.

**
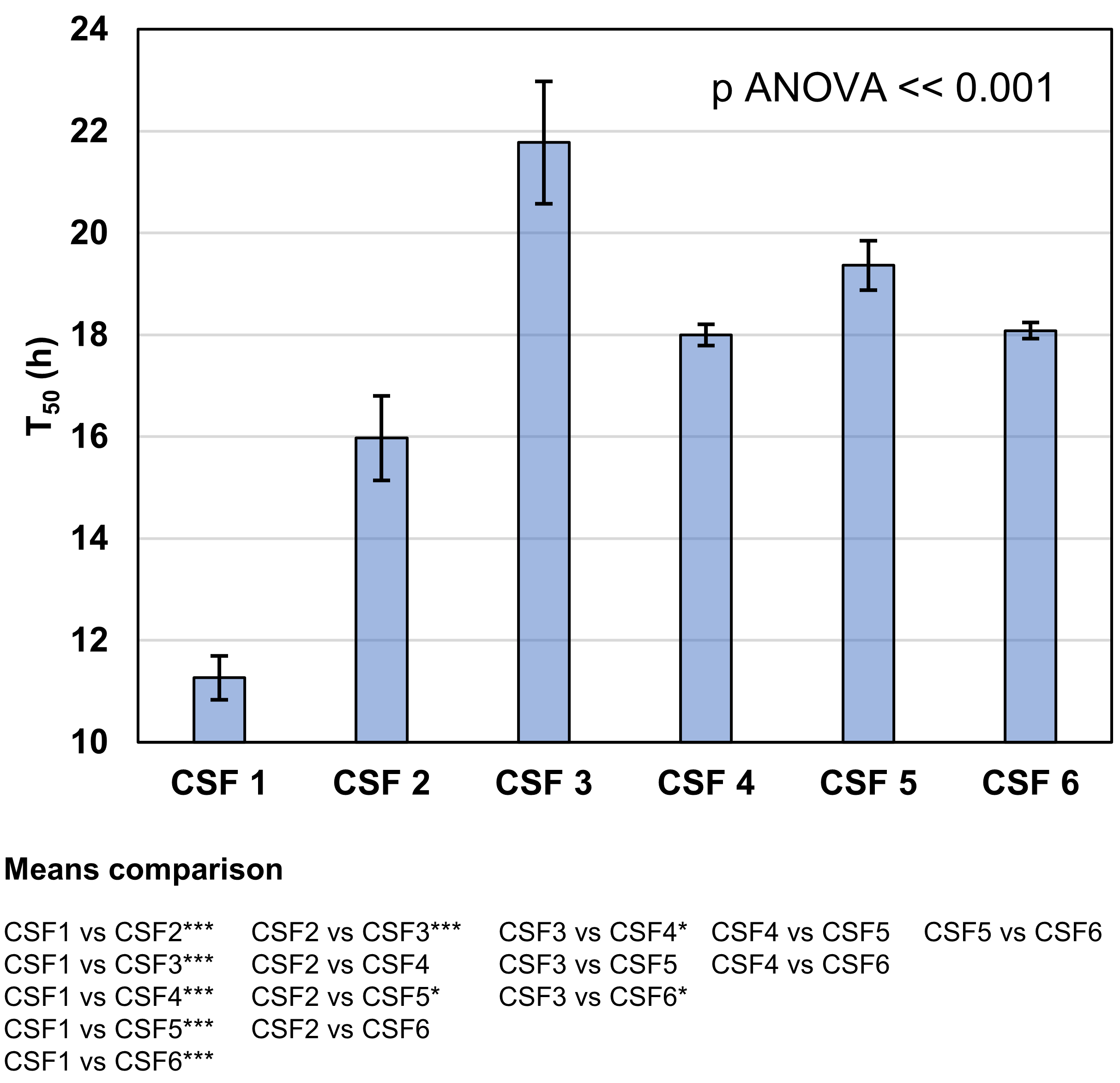
**

**Fig. S2.** **T_50_ measured in Six different human CSF samples spiked with 20 fg of seeds.** The measured T_50_ parameters were globally different, as assessed by one-way analysis of variance (ANOVA) and Fisher’s LSD post-hoc test for mean comparisons. *0.01<p<0.05; **0.001<p<0.01: ***p<0.001.

**
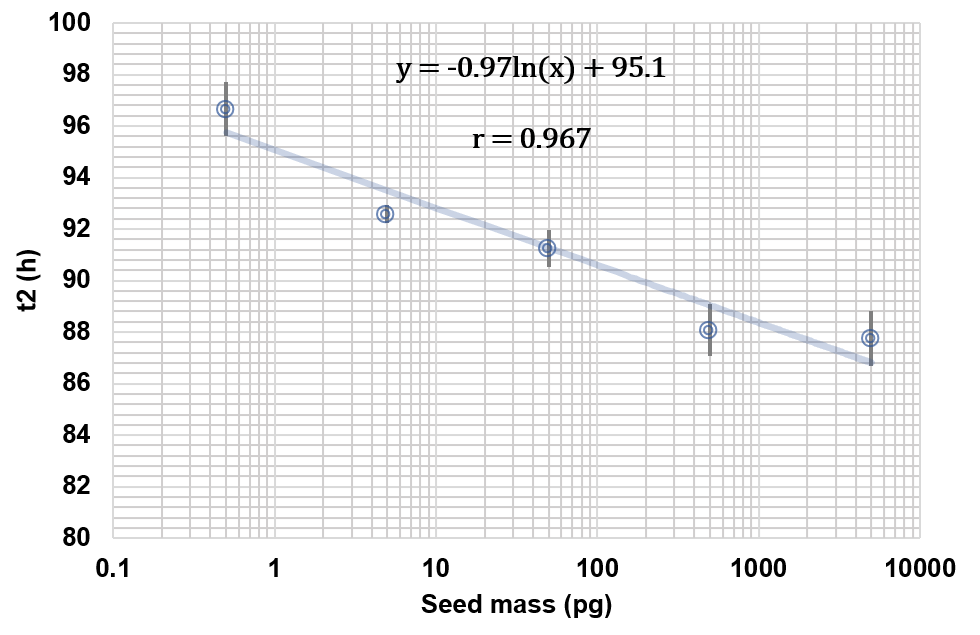
**

**Fig. S3. Quantitative regression analysis of the seed masses in the absence of human CSF.** Measured t2 parameters for the seeded experiment vs the quantity of seeds added; the horizontal axis is displayed in log10 scale. The t2 values displayed result from the average of three replicates, error bars reflect the standard deviation of the mean value. The data were fitted with a natural log function, the correlation between t2 and the added seed masses was assessed by means of Pearson’s correlation coefficient (r).


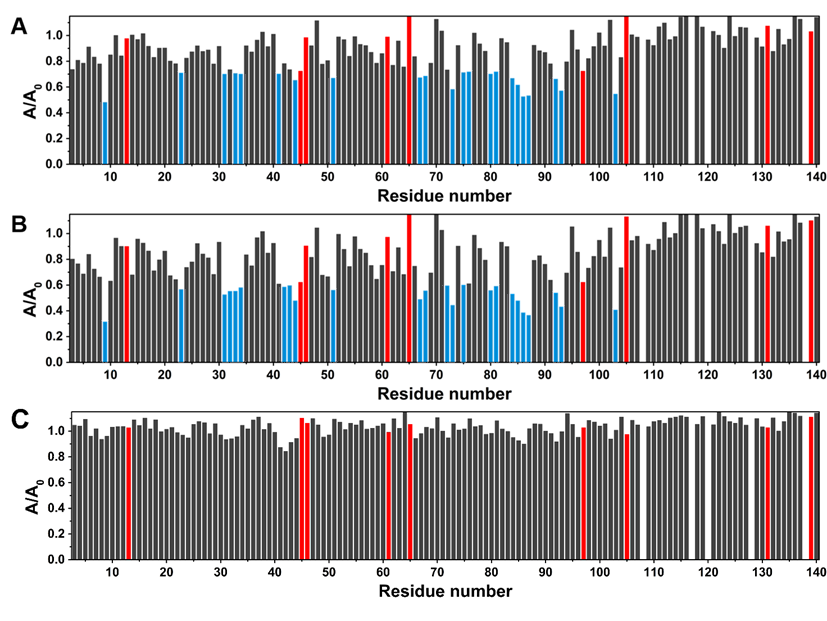


## Fig. S4. NMR titrations of α-syn with CSF fractions. Intensity decreases of the signals of two-dimensional (2D) ^15^N–^1^H HSQC experiments acquired at 950 MHz at T = 283 K on ^15^N labelled α-syn (100 μM) in PBS after the addition of: (A) whole pooled CSF in PBS, (B) < 3 kDa CSF fraction in PBS and (C) > 100 kDa CSF fraction in PBS. The residues experiencing the largest decreases in signal intensity (smaller by one or more standard deviations with respect to the average value) are highlighted in light blue. The intensity ratios corresponding to overlapping peaks are highlighted in red (their values were not considered in the calculation of the average decreases and standard deviations).

**
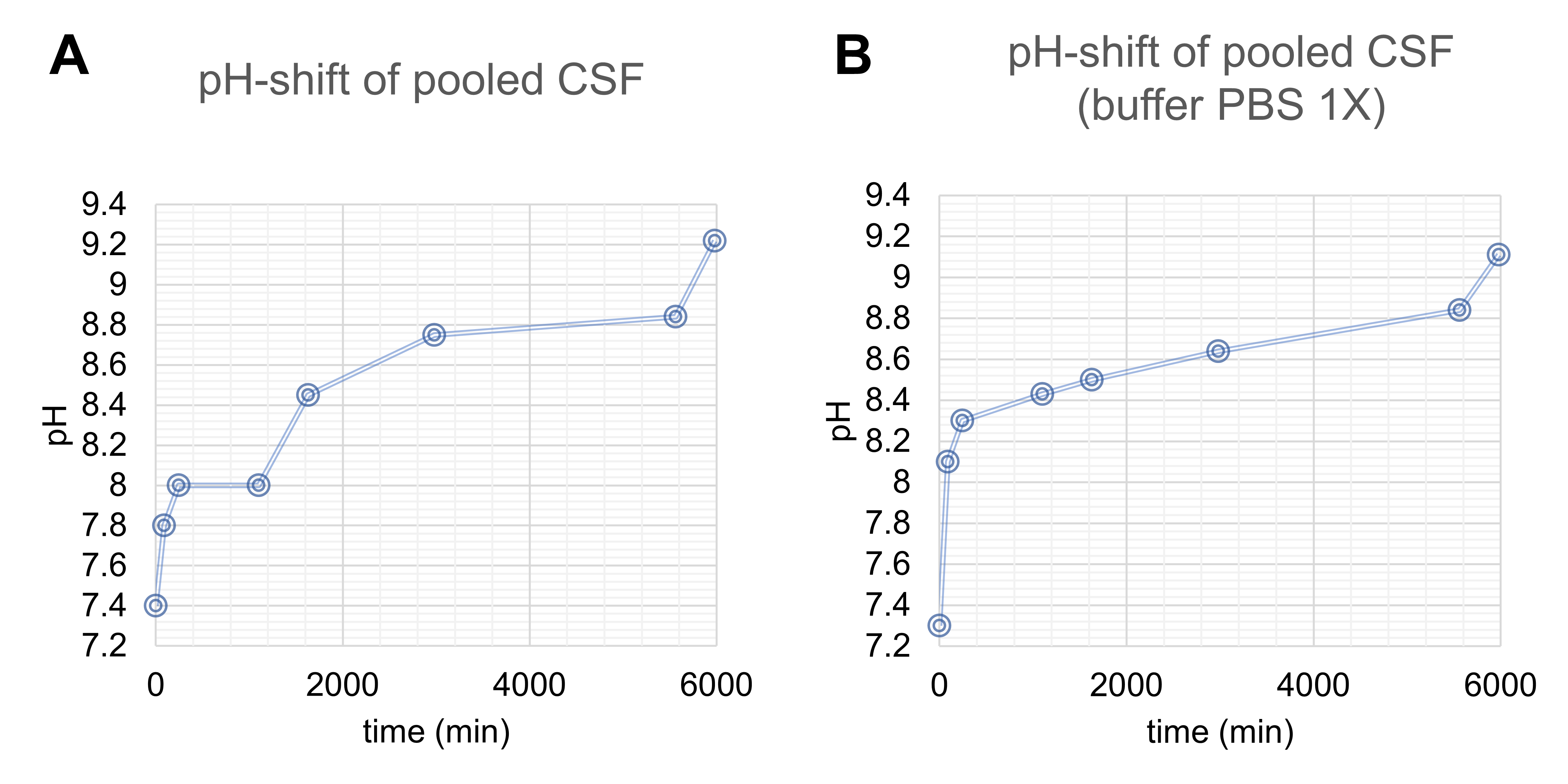
**

***Fig. S5. CSF pH drift.*** The pH change due to the exposure of CSF to air was monitored over time in 500 μL of undiluted pooled CSF (A) and in the presence of PBS (400 μL CSF + 200 μL PBS 3x) in polypropylene vials with a Thermo Scientific Orion pH-meter equipped with a glass 6 mm diameter pHenomenal MIC 220 Micro electrode. Right before each measurement, the sample was vortexed for 20 sec and left open to air for another 20 sec.


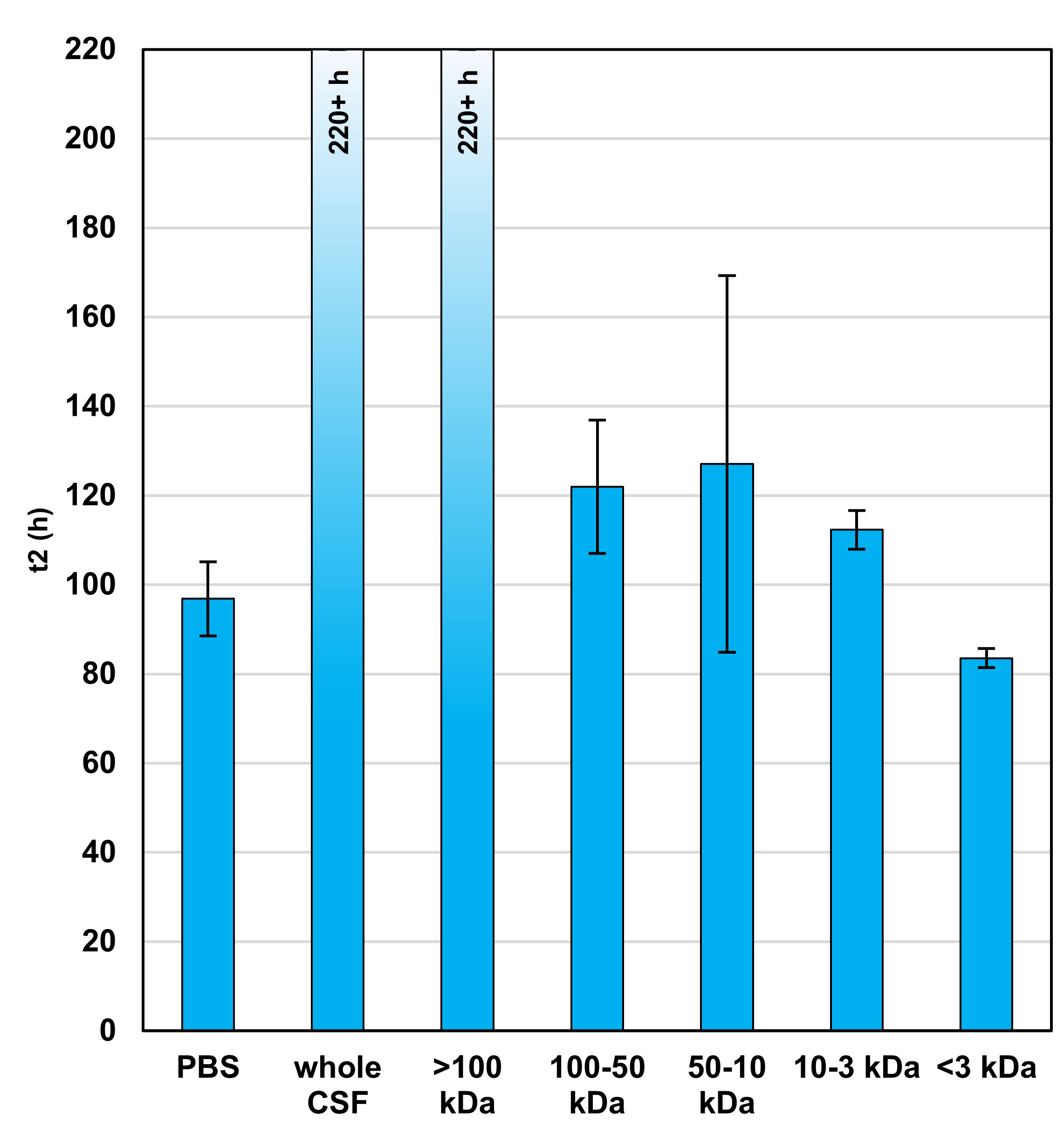


**Fig. S6.** **Different CSF fractions differently affect α-syn aggregation.** Mean fitted t2 parameters of samples with 40 μl of PBS/CSF fractions. The values displayed result from the average of three replicates with error bars reflecting the SEM. For whole CSF and the >100 kDa fraction the total duration of the experiment is shown due to the absence of appreciable aggregation.


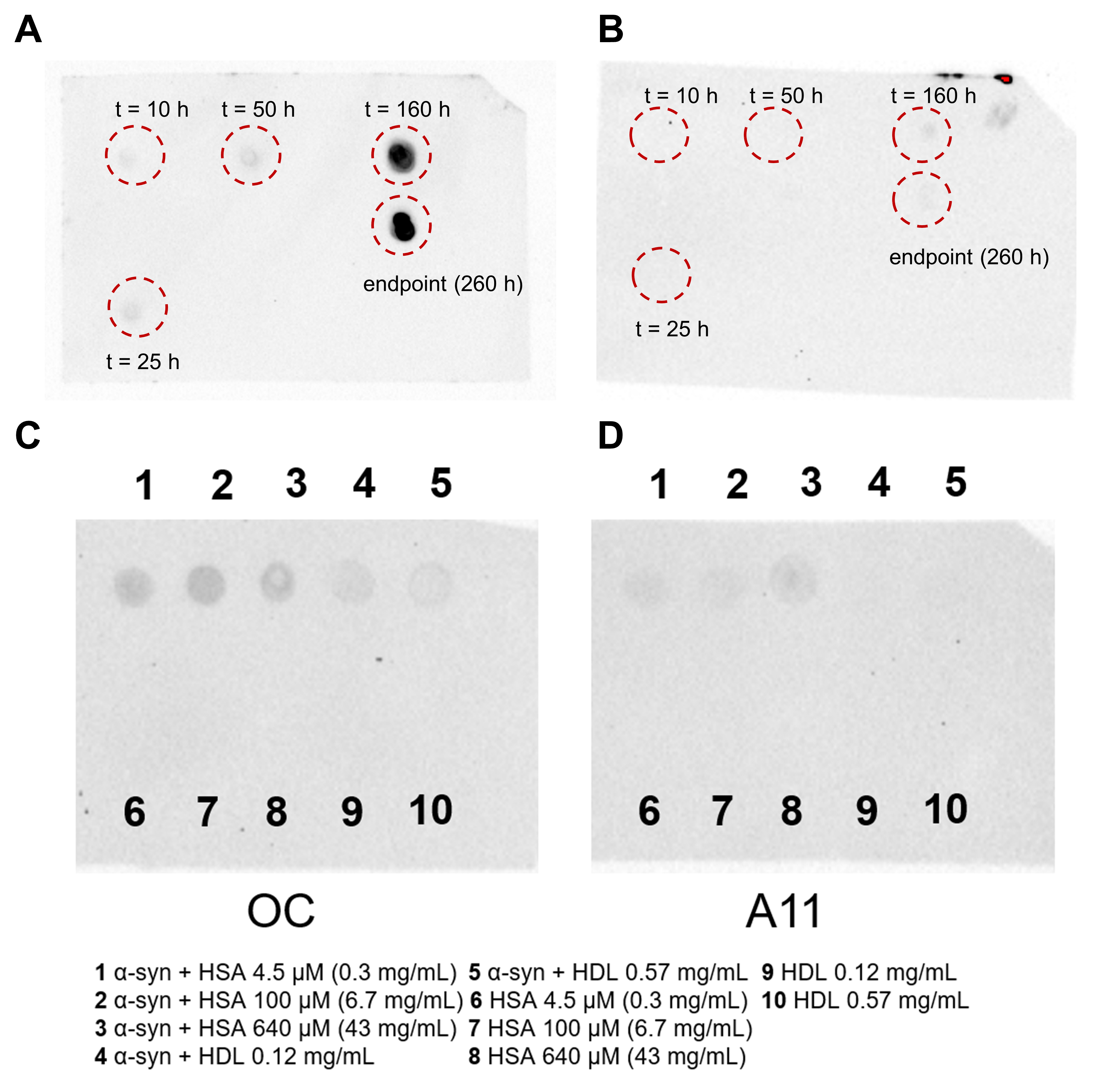


***Fig. S7. Raw images of the dot-blot assays.*** A-B) Native images of the dot-blot assay performed on α-syn alone replicates at different timepoints with OC (A) and A11 (B) conformational antibodies. C-D) Native image of the dot-blot assay performed on the HSA and HDL containing samples with OC (C) and A11 (D) conformational antibodies.


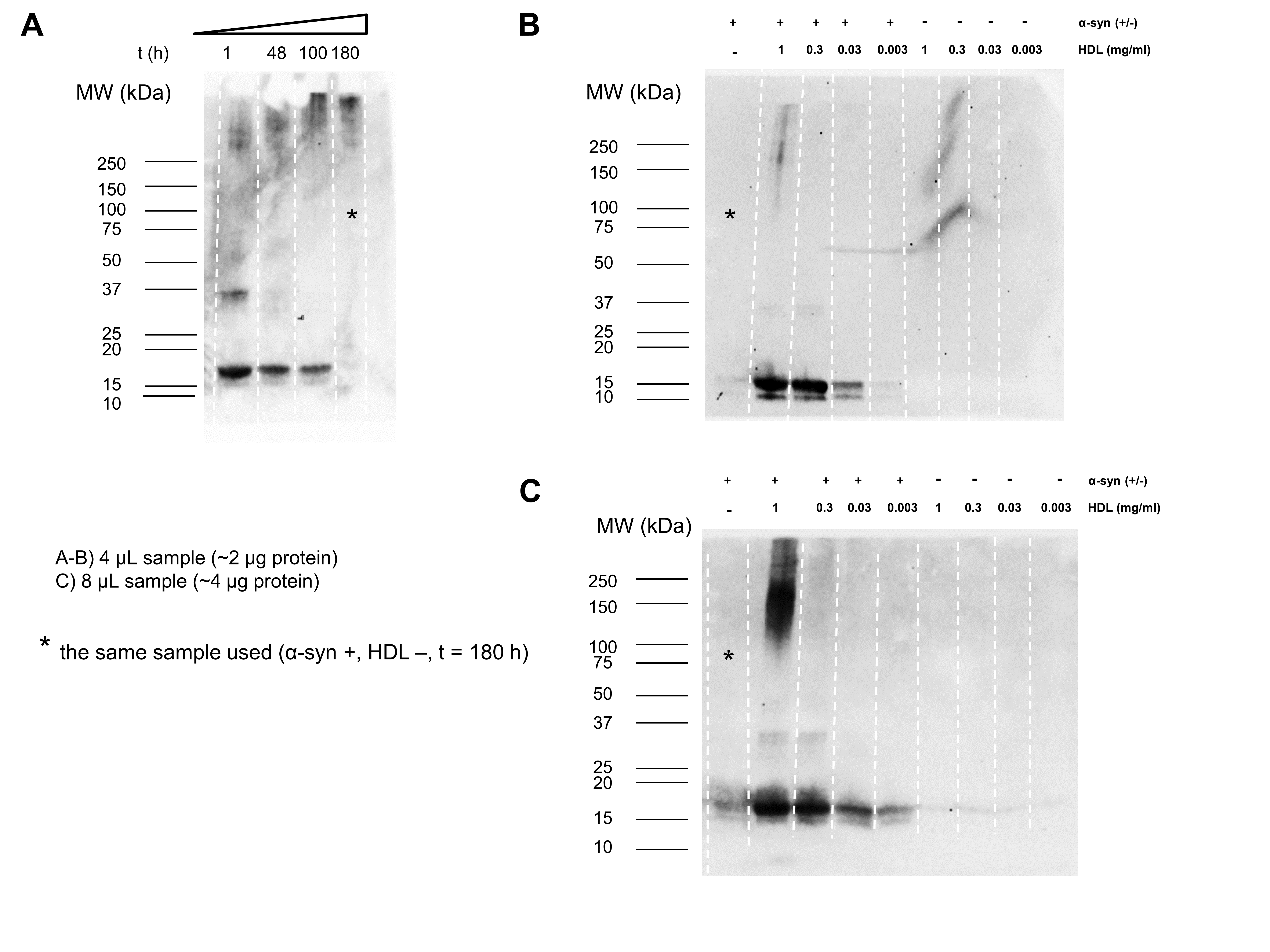


## Fig. S8. WB experiments to track α-syn aggregation in the presence of human HDL. A) α-Syn aggregation patterns in samples collected at different timepoints of the spontaneous aggregation process, was monitored by WB using Syn211 antibody (4-20% SDS-PAGE, 2 μg protein loaded). Monomeric α-syn decreases as t increases due to the formation of fibrils. B) In a similar way, a WB with Syn211 was performed on the reaction products obtained after 180 h, at different HDL concentrations with and without α-syn (exposure time 210 s). C) The experiment was then repeated by doubling the amount of sample loaded into the gel to better highlight the presence of oligomeric species (exposure time 30 s). Under these conditions, chosen to better visualize the signal at 150-200 kDa, the α-syn monomer bands at 1 and 0.3 mg/mL HDL may not quantitively reflect monomer concentration (overloaded lanes).

**
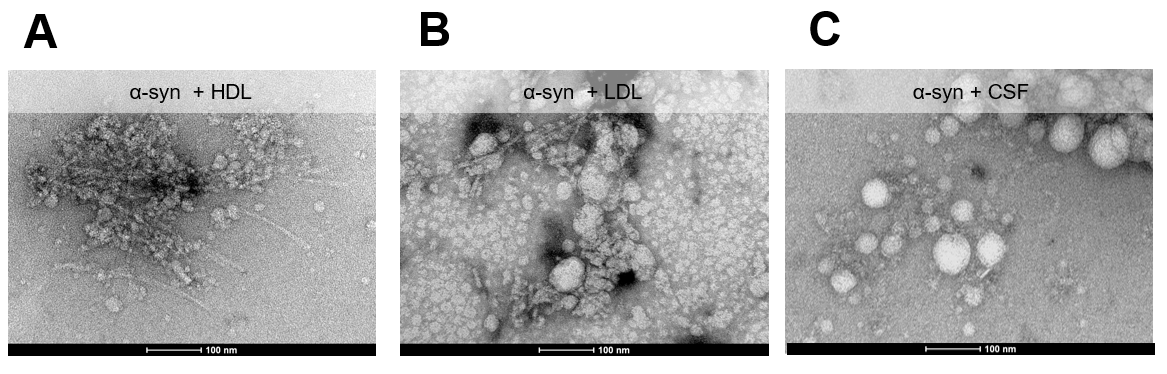
**



**Fig. S9. Representative TEM images of α-syn incubated with CSF.** Representative TEM images obtained by analyzing samples obtained by the co-incubation of α-syn 0.7 mg/mL at 37 °C with pooled human CSF (1:5 ratio with respect to total reaction volume). Samples were subjected to cycles of incubation (13 min) and shaking (double-orbital, 2 min) at 500 rpm.

**
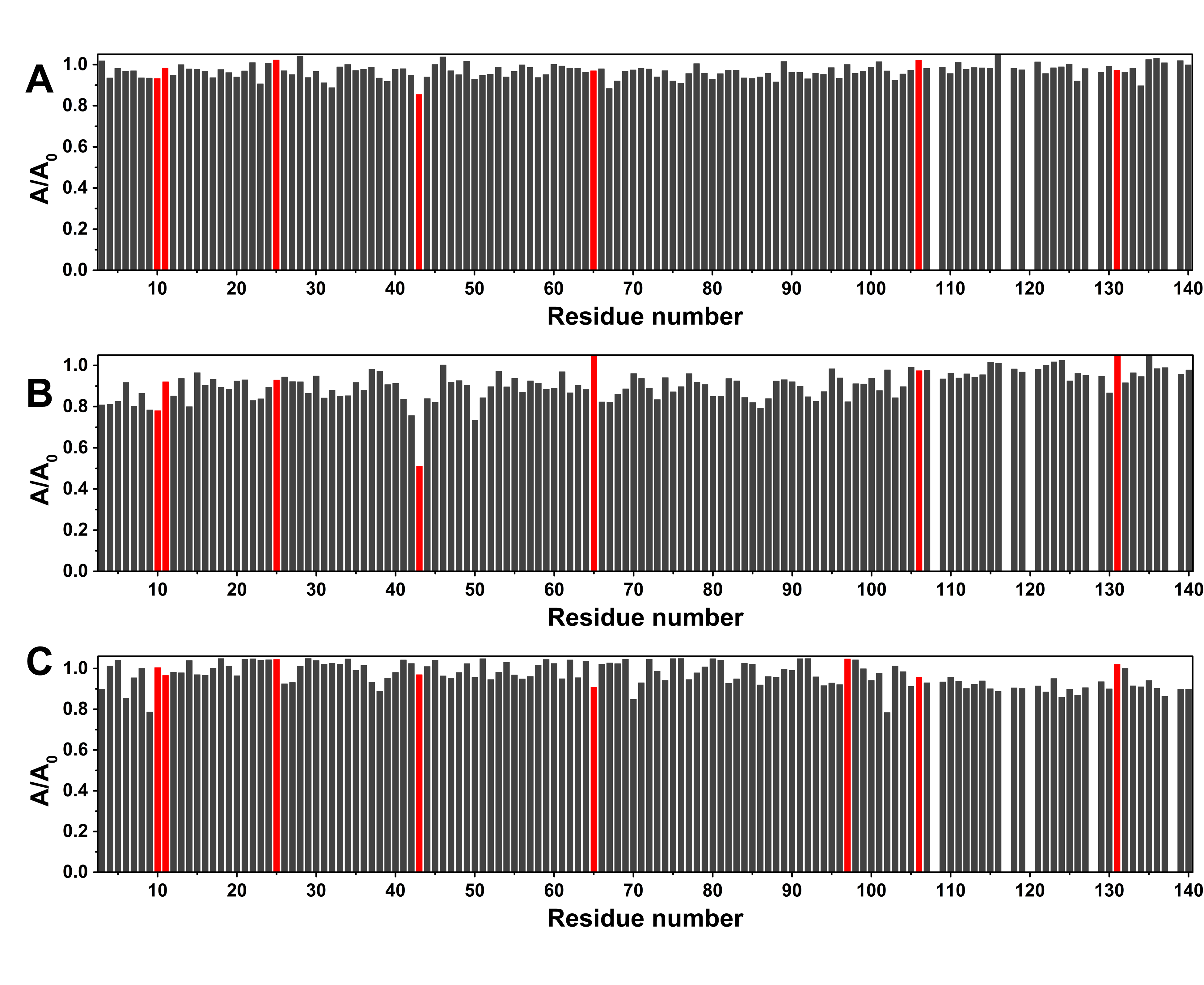
**

**Fig. S10. NMR titrations of α-syn with HDL, LDL and TTR. A)** Intensity decreases of the signals of two-dimensional (2D) ^15^N–^1^H HSQC experiments acquired at 950 MHz at T = 283 K on ^15^N labelled α-syn (100 μM) in PBS after the addition of 0.57 mg/mL serum-derived HDL. The intensity ratios corresponding to overlapping peaks are highlighted in red. B) Intensity decreases of the signals of two-dimensional (2D) ^15^N–^1^H HSQC experiments acquired at 950 MHz at T = 283 K on ^15^N labelled α-syn (100 μM) in PBS after the addition of 1 mg/mL serum-derived LDL. C) Intensity decreases of the signals of two-dimensional (2D) ^15^N–^1^H HSQC experiments acquired at 950 MHz at T = 283 K on ^15^N labelled α-syn (100 μM) in PBS after the addition of 3 mg/mL TTR.


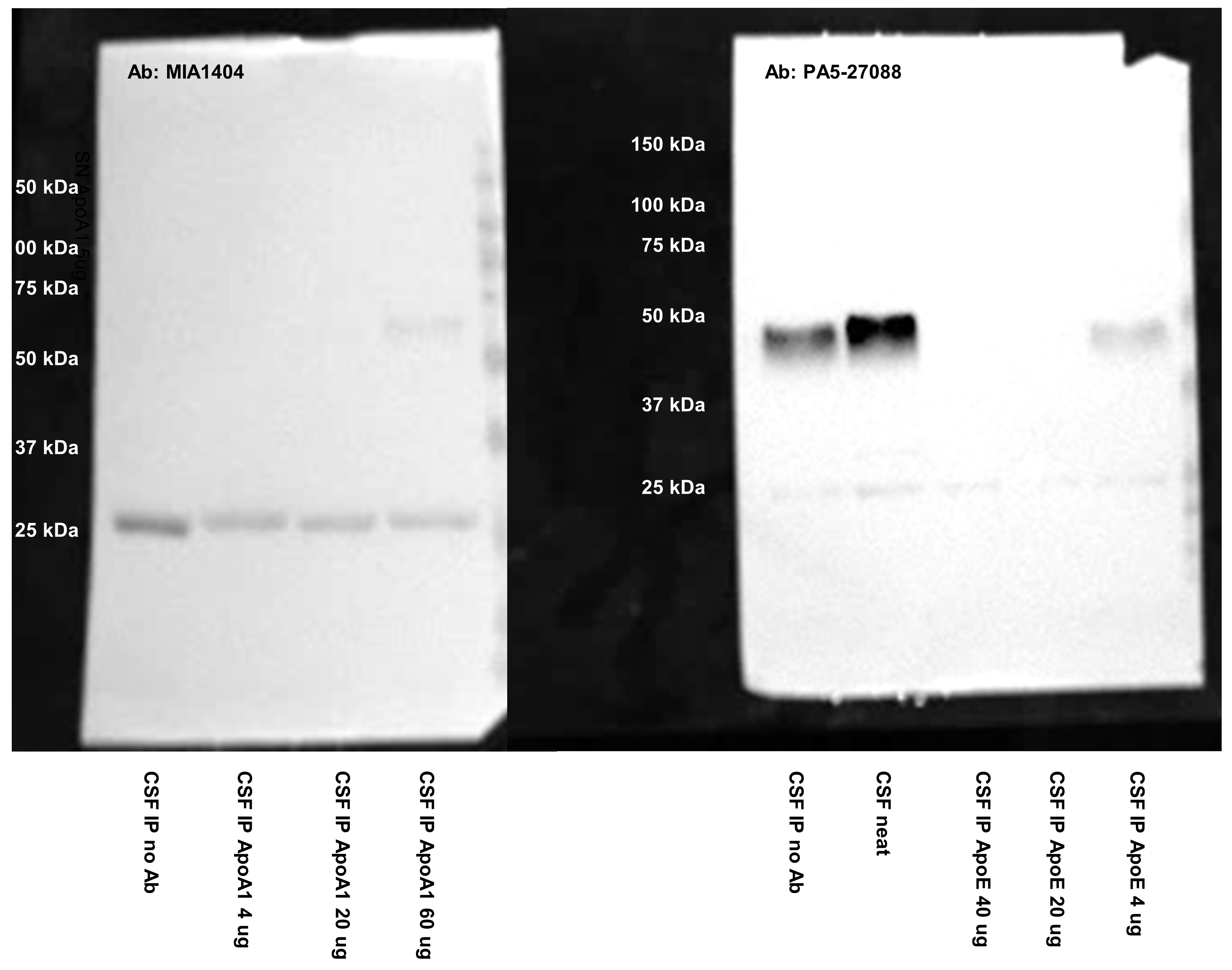


## Fig. S11. WB experiments performed on immunodepleted CSF. WB experiments were performed with anti-ApoA1 (MIA1404) and anti-ApoE (PA5-27088) antibodies on neat CSF and supernatants (400 μL CSF, 100 μL slurry) resulting from immunoprecipitation procedure performed using different quantities of the same antibodies (IP CSF samples), immunoprecipitation performed without antibodies (CSF IP no Ab). The conditions relative to IP CSF ApoA1 60 µg and IP CSF ApoE 20 µg were then selected for Protein aggregation assays.

**Supplementary Tables**

**Table S1. Concentration factors and final volumes of the CSF fractions.**

| **Sample number** | **MW range** | **Volume (mL)** | **Native concentration factor** | **Final concentration factor** |
| --- | --- | --- | --- | --- |
| 1 | whole CSF | 0.5 | 1 | 1 |
| 2 | >100 kDa | 0.5 | 8 | 5 |
| 3 | 50-100 kDa | 0.5 | 7 | 5 |
| 4 | 10-50 kDa | 0.5 | 6 | 5 |
| 5 | 3-10 kDa | 0.5 | 5 | 5 |
| 6 | <3 kDa | 2 | 1 | 1 |

**Table S2. Measured levels of the most abundant macromolecular CSF constituents by nLC-nESI HRMS/MS.** Albumin was the most abundant protein in whole CSF at its native concentration and in all concentrated fractions, although its level was similar to the one of apolipoproteins (apolip.) in the > 100 kDA fraction. TTR was abundant in the 100-50 kDa fraction. Apolipoproteins were mostly present in the >100 kDa fraction but relevant quantities were also detected in the 100-50 kDa and 50-10 kDa fractions while PGDS was mostly present in the 50-10 kDa fraction. Table cells are colored according to the relative concentration (emPAI x MW, proportional to the protein concentration in mg/mL) of each analyte in each concentrated CSF fraction, from blue (most abundant), to white (low levels or not detected, n.d.), the highest value of each analyte is highlighted in bold. The overall dilution/concentration factors (with respect to whole CSF) of the samples depicted are summarized in Table 3 in the Materials and Methods section.

| **Sample** | **whole CSF** | **>100 kDa** | **50-100 kDa** | **50-10 kDa** | **10-3 kDa** | **<3kDa** |
| --- | --- | --- | --- | --- | --- | --- |
| **Albumin (MW x emPAI)** | (520 ± 40) · 10^4^ | (220 ± 30) · 10^4^ | (1090 ± 130) · 10^4^ | (180 ± 130) · 10^4^ | (1.4 ± 0.4) · 10^4^ | n.d. |
| **TTR (MW x emPAI)** | (130 ± 40) · 10^4^ | (11 ± 4) · 10^4^ | (270 ± 100) · 10^4^ | (14 ± 1) · 10^4^ | n.d. | n.d. |
| **apolip. (MW x emPAI)** | (52 ± 15) · 10^4^ | (180 ± 10) · 10^4^ | (28 ± 6) · 10^4^ | (2.4 ± 0.6) · 10^4^ | n.d. | n.d. |
| **PGDS (MW x emPAI)** | (16 ± 4) · 10^4^ | (2.6 ± 1.6) · 10^4^ | (13 ± 2) · 10^4^ | (30 ± 14) · 10^4^ | n.d. | n.d. |

**Table S3. Mean fitted kinetic parameters and maximum fluorescence values (F_max_) of the analyzed samples.** Fitting was not possible for samples containing 0.3 and 1 mg/mL of HDL. Data are represented as mean ± SEM.

| **HDL (mg/mL)** | **F_max_**  **(a.u.)** | **2^nd^ plateau** | **1^st^ plateau** | **2^nd^ inflection** | **1^st^ inflection** |
| --- | --- | --- | --- | --- | --- |
|  |  | **A2 (a.u.)** | **A1 (a.u.)** | **t2 (h)** | **t1 (h)** |
| 0 | (31 ± 6) ∙ 10^3^ | (27 ± 5) ∙ 10^3^ | (12 ± 1.2) ∙ 10^2^ | 120 ± 11 | 39.1 ± 0.3 |
| 0.003 | (27 ± 4) ∙ 10^3^ | (22 ± 4) ∙ 10^3^ | (9.4 ± 1.7) ∙ 10^2^ | 135 ± 9 | 29.6 ± 1.3 |
| 0.03 | (35 ± 3) ∙ 10^3^ | (28 ± 2) ∙ 10^3^ | (3.3 ± 0.7) ∙ 10^2^ | 139 ± 7 | 30.2 ± 0.3 |
| 0.3 | (50 ± 2) ∙ 10 | n.d. | n.d. | > 215 | > 215 |
| 1 | (49 ± 3) ∙ 10 | n.d. | n.d. | > 215 | > 215 |

**Table S4**. **Summary of the final SAA outcome for PD47 and HC22 samples**. F_max_ values for each replicate and Final SAA outcome for PD47 and HC22 neat CSF samples (1x physiological concentration) and the same samples additioned with, 0.006 mg/mL (2x physiological concentration) and 0.024 mg/mL (5x physiological concentration) HDL or LDL; 0 mg/mL (1x), 0.004 mg/mL (2X) and 0.016 mg/mL (5x) TTR; 0 mg/mL (1x), 0.04 mg/mL (2x) and 0.16 mg/mL (5x) HSA. The outcome was categorized as: positive (+) when 3/3 replicates were determined positive by the probabilistic algorithm, inconclusive (?) when 2/3 replicates were determined positive by the probabilistic algorithm, and negative (-) when just 1/3 or 0/3 replicates were determined positive by the probabilistic algorithm.

| **Sample** | **F_max_1 (a.u)** | **F_max_2 (a.u)** | **F_max_3 (a.u)** | **(+/?/-)** |
| --- | --- | --- | --- | --- |
| PD47 neat | 51797 | 75341 | 37890 | + |
| PD47 2x HDL | 59834 | 73296 | 63038 | + |
| PD47 5x HDL | 3148 | 37277 | 45754 | ? |
| PD47 2x LDL | 61690 | 66693 | 58508 | + |
| PD47 5x LDL | 59274 | 14758 | 55548 | + |
| PD47 2x TTR | 41581 | 56848 | 54429 | + |
| PD47 5x TTR | 50889 | 56382 | 71270 | + |
| PD47 2x HSA | 45101 | 79406 | 53426 | + |
| PD47 5x HSA | 54536 | 43938 | 55022 | + |
| HC22 neat | 9345 | 126 | 129 | - |
| HC22 2x HDL | 126 | 128 | 134 | - |
| HC22 5x HDL | 130 | 134 | 137 | - |
| HC22 2x LDL | 133 | 136 | 134 | - |
| HC22 5x LDL | 6566 | 369 | 158 | - |
| HC22 2x TTR | 125 | 129 | 138 | - |
| HC22 5x TTR | 129 | 145 | 145 | - |
| HC22 2x HSA | 133 | 134 | 142 | - |
| HC22 5x HSA | 134 | 137 | 141 | - |
|  |  |  |  |  |
| 125 a.u. | 8000 a.u. |  |  | |

**SI References**

1. J. G. Cunniffe, S. Whitby-Strevens, M. H. Wilcox, Effect of pH changes in cerebrospinal fluid specimens on bacterial survival and antigen test results. *J Clin Pathol* **49**, 249–253 (1996).

2. A. Fura, T. W. Harper, H. Zhang, L. Fung, W. C. Shyu, Shift in pH of biological fluids during storage and processing: effect on bioanalysis. *Journal of Pharmaceutical and Biomedical Analysis* **32**, 513–522 (2003).

3. V. N. Uversky, J. Li, A. L. Fink, Evidence for a Partially Folded Intermediate in α-Synuclein Fibril Formation *. *Journal of Biological Chemistry* **276**, 10737–10744 (2001).

4. V. E. Shevchik, G. Condemine, J. Robert-Baudouy, Characterization of DsbC, a periplasmic protein of Erwinia chrysanthemi and Escherichia coli with disulfide isomerase activity. *The EMBO Journal* **13**, 2007–2012 (1994).

5. C. Huang, G. Ren, H. Zhou, C. Wang, A new method for purification of recombinant human α-synuclein in Escherichia coli. *Protein Expression and Purification* **42**, 173–177 (2005).

6. G. Bellomo, *et al.*, Dissecting the Interactions between Human Serum Albumin and α-Synuclein: New Insights on the Factors Influencing α-Synuclein Aggregation in Biological Fluids. *J Phys Chem B* **123**, 4380–4386 (2019).

7. K. M. Pate, R. M. Murphy, Cerebrospinal Fluid Proteins as Regulators of Beta-amyloid Aggregation and Toxicity. *Isr J Chem* **57**, 602–612 (2017).

8. E. R. Padayachee, *et al.*, Cerebrospinal fluid-induced retardation of amyloid β aggregation correlates with Alzheimer’s disease and the APOE ε4 allele. *Brain Res* **1651**, 11–16 (2016).

9. M. Shahnawaz, *et al.*, Development of a Biochemical Diagnosis of Parkinson Disease by Detection of α-Synuclein Misfolded Aggregates in Cerebrospinal Fluid. *JAMA Neurology* **74**, 163 (2017).

10. L. Concha-Marambio, M. Shahnawaz, C. Soto, Detection of Misfolded α-Synuclein Aggregates in Cerebrospinal Fluid by the Protein Misfolding Cyclic Amplification Platform. *Methods in molecular biology (Clifton, N.J.)* **1948**, 35–44 (2019).

11. J. Adler, H. A. Scheidt, K. Lemmnitzer, M. Krueger, D. Huster, N-terminal lipid conjugation of amyloid β(1–40) leads to the formation of highly ordered N-terminally extended fibrils. *Phys. Chem. Chem. Phys.* **19**, 1839–1846 (2017).

12. R. G. Morris, J. L. Evenden, B. J. Sahakian, T. W. Robbins, “Computer-aided assessment of dementia: Comparative studies of neuropsychological deficits in Alzheimer-type dementia and Parkinson’s disease” in *Cognitive Neurochemistry*, (Oxford University Press, 1987), pp. 21–36.

13. Y. Ishihama, *et al.*, Exponentially modified protein abundance index (emPAI) for estimation of absolute protein amount in proteomics by the number of sequenced peptides per protein. *Mol Cell Proteomics* **4**, 1265–1272 (2005).

14. K. Shinoda, M. Tomita, Y. Ishihama, emPAI Calc--for the estimation of protein abundance from large-scale identification data by liquid chromatography-tandem mass spectrometry. *Bioinformatics* **26**, 576–577 (2010).
